# Ultrasound‐Stimulated “Exocytosis” by Cell‐Like Microbubbles Enhances Antibacterial Species Penetration and Immune Activation Against Implant Infection

**DOI:** 10.1002/advs.202307048

**Published:** 2023-12-18

**Authors:** Weijun Xiu, Xiaoye Li, Qiang Li, Meng Ding, Yu Zhang, Ling Wan, Siyu Wang, Yu Gao, Yongbin Mou, Lianhui Wang, Heng Dong

**Affiliations:** ^1^ Nanjing Stomatological Hospital Affiliated Hospital of Medical School Nanjing University 30 Zhongyang Road Nanjing 210008 P. R. China; ^2^ Key Laboratory for Organic Electronics and Information Displays Jiangsu Key Laboratory for Biosensors Institute of Advanced Materials Jiangsu National Synergetic Innovation Centre for Advanced Materials Nanjing University of Posts and Telecommunications 9 Wenyuan Road Nanjing 210023 P. R. China

**Keywords:** bacterial biofilms, immune therapy, implant infection, microbubbles, ultrasound‐responsive drug delivery

## Abstract

Host immune systems serving as crucial defense lines are vital resisting mechanisms against biofilm‐associated implant infections. Nevertheless, biofilms hinder the penetration of anti‐bacterial species, inhibit phagocytosis of immune cells, and frustrate host inflammatory responses, ultimately resulting in the weakness of the host immune system for biofilm elimination. Herein, a cell‐like construct is developed through encapsulation of erythrocyte membrane fragments on the surface of Fe_3_O_4_ nanoparticle‐fabricated microbubbles and then loaded with hydroxyurea (EMB‐Hu). Under ultrasound (US) stimulation, EMB‐Hu undergoes a stable oscillation manner to act in an “exocytosis” mechanism for disrupting biofilm, releasing agents, and enhancing penetration of catalytically generated anti‐bacterial species within biofilms. Additionally, the US‐stimulated “exocytosis” by EMB‐Hu can activate pro‐inflammatory macrophage polarization and enhance macrophage phagocytosis for clearance of disrupted biofilms. Collectively, this work has exhibited cell‐like microbubbles with US‐stimulated “exocytosis” mechanisms to overcome the biofilm barrier and signal macrophages for inflammatory activation, finally achieving favorable therapeutic effects against implant infections caused by methicillin‐resistant *Staphylococcus aureus* (MRSA) biofilms.

## Graphical abstract

Under ultrasound (US) stimulation, erythrocyte membrane fragments encapsulated microbubbles loaded with hydroxyurea (EMB‐Hu) can contract in an oscillatory manner for agent release. Such US‐stimulated “exocytosis” by EMB‐Hu can disrupt biofilm structure by the generation of microstream, enhance the penetration of antibacterial species  within biofilms, and also activate macrophages for efficient elimination of residual  biofilms during implant infection.

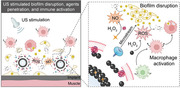

## 1 Introduction

Biomedical implants, such as orthopedic implants, dental implants, catheters, and a variety of other devices, have revolutionized medicine, but they also pose an increased risk of infection.^[^ ^1^ ^]^ To date, implant infection has gradually become the most common and serious complication associated with the use of biomaterials.^[^ ^2^ ^]^ In the USA, ≈25.6% of healthcare‐associated infections were related to implant infection.^[^ ^3^ ^]^ *Staphylococcus aureus* is one of the most frequently occurring bacteria during implant infections, and a high level of competence is demonstrated in the production of biofilms on implant surfaces by secreting extracellular polymeric substances (EPS) to encapsulate themselves tightly, thereby protecting them from the attack of bactericides or host immune defense.^[^ ^3^, ^4^ ^]^ Although antibiotics have been commonly used for the treatment of implant infections, the emergence of bacterial resistance hampers their durability.^[^ ^5^ ^]^ The host immune system has a significant impact on bacteria elimination, and can hardly develop bacterial resistance. On the one hand, immune cells can secrete anti‐bacterial species, such as reactive oxygen species (ROS) and nitric oxide (NO), to kill bacteria. On the other hand, phagocytic immune cells can engulf bacteria to inactivate bacteria.^[^ ^2^, ^6^ ^]^ However, owing to the protection of EPS, biofilms can not only restrict the penetration of anti‐bacterial species for inhibiting their antibacterial efficiency but also prevent the interaction of immune cells with bacteria embedded within them and result in “frustrated phagocytosis” by phagocytes.^[^ ^3^, ^7^ ^]^ In addition, biofilms can alter macrophage polarization from the classic pro‐inflammatory (M1) into the anti‐inflammatory (M2) phenotype to inhibit inflammatory responses of the host immune system, which further limits the recruitment of immune cells to infected sites and impedes biofilm clearance.^[^ ^3^, ^8^ ^]^ The pathogen clearance effect of the host immune system is therefore seriously hindered during biofilm infections.

To explore the mechanisms by which natural reaction networks regulate the behavior, operation, and communication of natural cells, most biocompatible artificial constructs with simple membrane‐bound cell‐like structures have been developed to possess the minimal functions of living cells.^[^ ^9^ ^]^ These synthetic cell‐like constructs are typically created using colloidosomes, polymersomes, liposomes, or proteinosomes as semi‐permeable membrane structures, and have successfully exhibited a range of collective protocell behaviors, such as migration,^[^ ^10^ ^]^ phagocytosis,^[^ ^11^ ^]^ exocytosis,^[^ ^12^ ^]^ and signaling.^[^ ^13^ ^]^ As a result of their cell‐like characteristics, such cell‐like constructs are suitable candidates for biologically and artificially combined algorithms, and provide bright applications in biomedicine and bioengineering. Currently, the majority of micrometer‐scale structures featuring cell‐like constructs have been designed to treat diverse ailments. For example, hydrogel‐encapsulated proteinosomes have been used for the intracellular killing of tumor cells,^[^ ^14^ ^]^ the multi‐compartmentalized lipid vesicles fabricated as synthetic beta cells exploited for fusion‐mediated insulin secretion against diabetes,^[^ ^12^ ^]^ and the erythrocyte membrane‐encapsulated coacervate protocells capable of catalytically generating NO for blood vessel vasodilation.^[^ ^15^ ^]^ As the pathogen clearance capability of immune cells is seriously restricted during biofilm infections, developing a novel cell‐like construct, which can overcome biofilm structure barriers and normalize the bacterial inactivation function of immune cells, may provide a prosing opportunity against biofilm‐associated implant infections.

**Figure 1:**
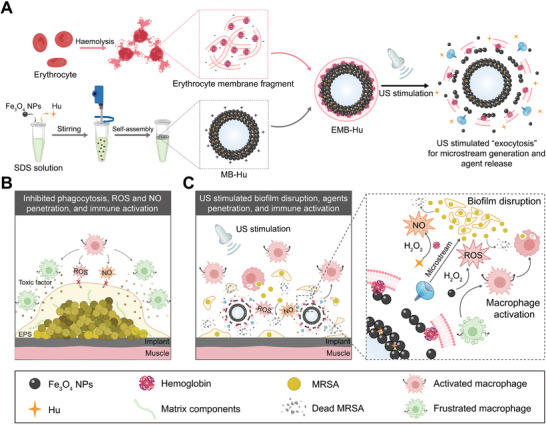
US‐stimulated “exocytosis” by EMB‐Hu enhances agent penetration and macrophage activation for the efficient elimination of MRSA biofilm. A) Schematic illustration of the preparation process of EMB‐Hu (SDS: sodium dodecyl sulfate, Hu: hydroxyurea) and the US‐stimulated microstream generation and agents release in EMB‐Hu. B) Formation of MRSA biofilms can prevent macrophage‐generated antibacterial species penetration, restrict phagocytosis effects of macrophage, and frustrate macrophage activation. C) Under US stimulation, EMB‐Hu can contract in an oscillatory manner for agent release, which as an “exocytosis”‐like pattern can not only disrupt biofilm structure and enhance antibacterial species (ROS and NO) penetration within the biofilm but also activate macrophages for efficient elimination of disrupted MRSA biofilms during implant infection.

In this work, we used ferriferous oxide nanoparticles (Fe_3_O_4_ NPs) fabricated microbubbles (MBs) as the basis to fabricate the cell‐like construct with US‐responsive capability for biofilm disruption and signaling‐elevated immune activation, therefore examining efficient biofilm elimination for the treatment of methicillin‐resistant *S. aureus* (MRSA) biofilm induced implant infection. Microbubbles with microscale spherical structures are commonly stabilized by polymer, protein, or liposome shells with a gas core. Owing to their specific acoustic properties, microbubbles were widely used as drug‐delivery systems for treating various diseases.^[^ ^16^ ^]^ Here, we developed cell‐like MBs with a membrane‐bound spherical structure by the self‐assembly of erythrocyte membrane fragments on the surface of Fe_3_O_4_ NPs fabricated MBs, and loaded with hydroxyurea (Hu) to form EMB‐Hu. The encapsulation of erythrocyte membrane fragments can not only enhance the stability of MBs, but facilitate the generation of NO with the reaction of Hu. Under low‐intensity US stimulation, EMB‐Hu entered a stable oscillation manner and induced the Hu and Fe_3_O_4_ NPs to “spit” away from EMB‐Hu like the cell exocytosis manner (**Figure** **1**A). Such US‐stimulated “exocytosis” by EMB‐Hu can both disrupt MRSA biofilm structure and facilitate penetrations of Hu and Fe_3_O_4_ NPs  within biofilms, further elevating the diffusion of catalytically generated NO and ROS inside biofilms for enhanced bacterial inactivation. In addition, the released Fe_3_O_4_ NPs can act as “signaling molecules” to polarize macrophages to the pro‐inflammatory phenotype to eliminate the disrupted biofilm. As proof of concept, we demonstrated that US‐stimulated “exocytosis” by EMB‐Hu can disrupt MRSA biofilms, enhance the anti‐bacterial species (ROS and NO) penetration within biofilms, and controllably elevate the inflammatory response of macrophages for efficient MRSA biofilm elimination both in vitro and in vivo, which compensated for the deficiency of frustrated immune cell functions during biofilm infections (Figure 1B,C).

## 2 Results

### 2.1 Synthesis and Characterization of EMB‐Hu

**Figure 2:**
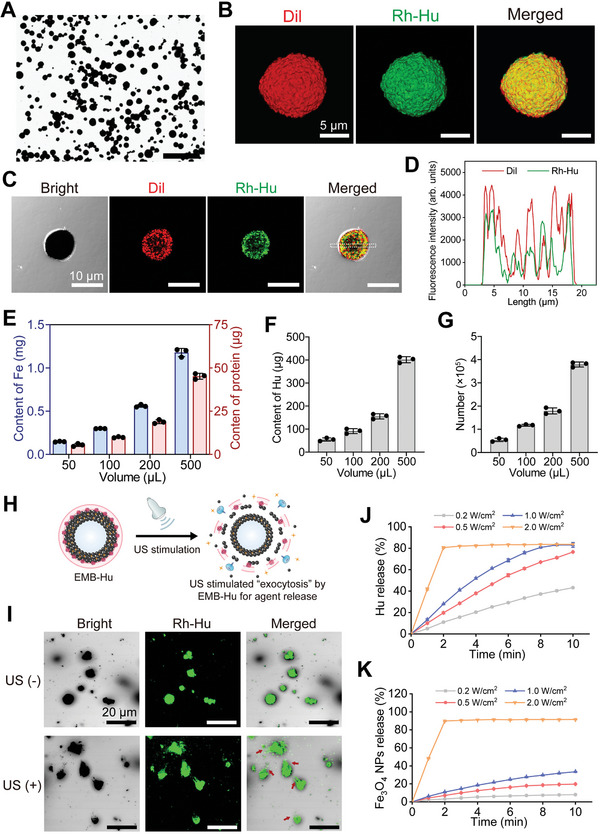
Preparation and characterization of EMB‐Hu. A) Bright‐field microscopy image of EMB‐Hu. Scale bar is 100 µm. B) 3D confocal laser scanning microscope (CLSM) images of EMB‐Hu. (Red: Dil, green: Rh‐Hu). C) CLSM image of EMB‐Hu and D) fluorescence profile of Dil (red) and Rh‐Hu (green) in EMB‐Hu. E) Quality of Fe, protein, F) Hu, and G) number of EMBs in different volumes of EMB‐Hu dispersions (*n* = 3, means ± SD). H) Schematic illustration of US‐stimulated “exocytosis” by EMB‐Hu. **I**) CLSM images of EMB‐Hu encapsulated in ultrasonic couplants with or without US stimulation (1 MHz, 0.5 W cm^−2^, 50% amplitude, 5 min). Red arrows indicate the generation of the microstream. J) Cumulative release profiles of Hu and K) Fe_3_O_4_ NPs from EMB‐Hu under US stimulation with different powers (1 MHz, 50% amplitude, *n* = 3, means ± SD).

The preparation of drug‐loaded MBs was performed according to our previous works.^[^ ^16^, ^17^ ^]^ Briefly, sodium dodecyl sulfate (SDS), hydroxyurea (Hu), and Fe_3_O_4_ NPs (≈150 nm size, Figure S1, Supporting Information) were mixed and agitated by using a homogenizer for 3 min to form Hu‐loaded MBs (MB‐Hu) (Figure S2, Supporting Information). The size of the as‐prepared MB‐Hu was ≈11.78 µm, with an ≈0.31 µm thickness of Fe_3_O_4_ NPs layers (Figures S3 and S4, Supporting Information). For further encapsulation of the erythrocyte membrane fragments, the negatively charged erythrocyte membrane fragments were mixed with positively charged MB‐Hu and incubated for 12 h at 4 °C to finally prepare erythrocyte membrane fragments encapsulated MB‐Hu (EMB‐Hu). As illustrated in **Figure** **2**A, EMB‐Hu retains its spherical morphology. After being coated with negatively charged erythrocyte membrane fragments, the zeta potential of EMB‐Hu changed from 14.5 mV (MB‐Hu) to −8.3 mV (EMB‐Hu) (Figure S5, Supporting Information). To confirm the successful loading of Hu and encapsulation of the erythrocyte membrane fragments in EMB‐Hu, rhodamine B‐labeled Hu (Rh‐Hu) and 1,1′‐dioctadecyl‐3,3,3′,3′‐tetramethylindocarbocyanine perchlorate (Dil, for staining erythrocyte membrane fragments) were used to label EMB‐Hu. As shown in Figure 2B–D and Figure S6, Supporting Information, an obvious fluorescence signal of Rh‐Hu and Dil can be observed on the surface of EMB‐Hu, while there is no fluorescence signal of Dil in MB‐Hu. SEM images of EMB‐Hu indicated the successful encapsulation of the erythrocyte membrane fragments on the surface of EMB‐Hu (Figure S7, Supporting Information). Besides, the elemental mapping images of EMB‐Hu show the uniform distribution of N and C in EMB‐Hu, and N in MB‐Hu, while no N and C signals can be observed in MBs (Figure S8, Supporting Information). Gel electrophoresis experiments indicated that the erythrocyte membrane fragment (subunit band at 14.4 kDa for hemoglobin) was present in EMB‐Hu (Figure S9, Supporting Information).^[^ ^15^ ^]^ All results indicated the successful loading of Hu and encapsulation of erythrocyte membrane fragments in EMB‐Hu. By counting the number of EMB‐Hu in different volumes of solutions, the amounts of Fe, protein, and Hu were determined to be 0.32 × 10^−8^, 0.25 × 10^−9^, and 0.95 × 10^−9^ g per EMB‐Hu, respectively (Figure 2E–G). When the feeding ratio for the amount of Fe_3_O_4_ NPs and Hu was 5:1, the loading efficiency of Hu was ≈5.3% (Figure S10, Supporting Information).

According to our previous work, low‐intensity US can stimulate Fe_3_O_4_ NPs‐shelled MBs into stable oscillation to act in an “exocytosis” manner for the gradual release of the loaded agents (Figure 2H).^[^ ^16b^ ^]^ In addition, US‐stimulated stable oscillation of microbubbles (MBs) combined with the inertia of the surrounding liquid can cause a rush‐in of fluid toward the MBs and finally induce the generation of microstream, which may disrupt the fortress‐like biofilm structure and promote penetration of agents within biofilms.^[^ ^16^, ^17^, ^18^ ^]^ As illustrated in Figure 2I–K, EMB‐Hu can generate a microstream under relatively low‐intensity US stimulation (0.5 W cm^−2^), and gradually release Hu and Fe_3_O_4_ NPs. The release of Hu and Fe_3_O_4_ NPs was increased with increasing US power. When the power of US increased to 2.0 W cm^−2^, most Hu and Fe_3_O_4_ NPs were quickly released with only 2 min of US stimulation, which may be attributed to the power of US exceeding the cavitation threshold of MBs to induce MB collapse (Figure S11, Supporting Information).^[^ ^16b^ ^]^

The peroxidase (POD)‐like catalytic activity of EMB‐Hu was evaluated by using 3,3′,5,5′‐tetramethylbenzidine (TMB) as a substrate.^[^ ^19^ ^]^ The erythrocyte membrane fragments showed obvious POD‐like catalytic activity owing to the presence of hemoglobin (Figure S12, Supporting Information). Fe_3_O_4_ NPs also showed POD‐like catalytic activity. After the formation of MBs, the POD‐like catalytic activity of EMB‐Hu was decreased compared with that of Fe_3_O_4_ NPs, which may be because of the inhibited catalytic site of Fe_3_O_4_ NPs inner MB shells.^[^ ^17^ ^]^ After US stimulation, the release of Fe_3_O_4_ NPs can further enhance the POD‐like catalytic activity of EMB‐Hu. The NO generation capacity of EMB‐Hu was further studied by using the Greiss reagent colorimetric assay. Previous work indicated that the clinically used drug hydroxyurea can be catalytically oxidized to form NO by horseradish peroxidase (HRP) and H_2_O_2_.^[^ ^20^ ^]^ As the hemoglobin in the erythrocyte membrane fragments and Fe_3_O_4_ NPs have POD‐like catalytic activity, we assume that the Fe_3_O_4_ NPs or erythrocyte membrane fragments can catalyze NO generation from Hu. As illustrated in Figure S13, Supporting Information, Hu and EMBs showed limited NO generation with the addition of H_2_O_2_. MB‐Hu and EMB‐Hu can slightly generate NO with the addition of H_2_O_2_. After US stimulation, the generation of NO by EMB‐Hu was increased, suggesting the US‐stimulated NO generation capability of EMB‐Hu under H_2_O_2_ conditions.

For further biomedical application, the hemolytic activity, cytotoxicity, and stability of EMB‐Hu were studied. The erythrocyte hemolysis ratio of MB‐Hu was ≈10.4% when the concentration of Fe_3_O_4_ NPs was 2 mg mL^−1^, while that of EMB‐Hu was 6.8% at the same concentration (Figure S14, Supporting Information), suggesting that the encapsulation of the erythrocyte membrane fragments can reduce the hemolysis ratio of MB‐Hu. When the concentration of Fe_3_O_4_ NPs in EMB‐Hu reached 2 mg mL^−1^, the viability of human oral keratinocytes (HOK) was above 85%, indicating the EMB‐Hu in this concentration showed a certain degree of cytotoxicity (Figure S15, Supporting Information). To ensure the biosafety of EMB‐Hu used in this work, the dose of EMB‐Hu in 500 µg mL^−1^ that showed a low level of hemolysis ratio (3.7%) and cytotoxicity (7.4%) was chosen in vitro experiments. In addition, the MB‐Hu showed poor stability after incubation with PBS or DMEM containing 10% FBS, while more than 90% of EMB‐Hu remained stable after incubation with PBS or DMEM containing 10% FBS for 24 h (Figure S16), indicating that the encapsulation of the erythrocyte membrane fragments can enhance the stability of EMB‐Hu in the physiological environment.

### 2.2 US‐Stimulated Biofilm Disruption and Antibacterial Species Penetration In Vitro

**Figure 3:**
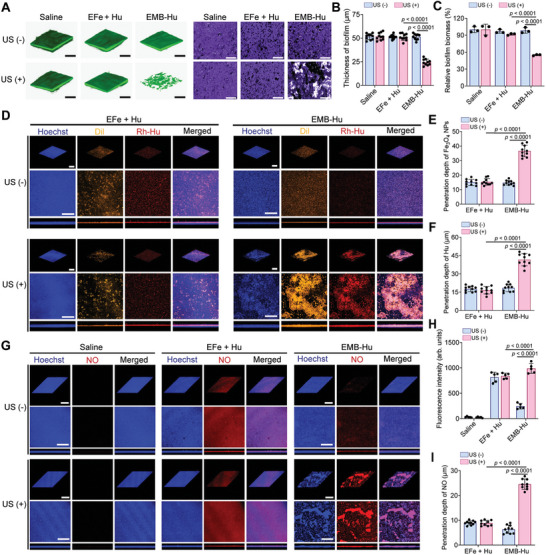
MRSA biofilm disruption enhances antibacterial species penetration in vitro. A) The 3D CLSM images of biofilm stained by Calcein‐AM (left) and microscope images of MRSA biofilm stained by crystal violet (right) in different groups (Fe_3_O_4_ NPs: 500 µg mL^−1^; Hu: 150 µg mL^−1^; US: 1 MHz, 0.5 W cm^−2^, 50% amplitude, 10 min). Scale bar is 200 µm. B) Thickness of biofilms in different groups calculated from (A) (*n* = 10, means ± SD). C) Relative biofilm biomass in different groups (*n* = 3, means ± SD). D) 3D CLSM images of biofilm after incubation with EFe + Hu and EMB‐Hu stained by Dil and Rh‐Hu in different conditions. Scale bar is 200 µm. E) Penetration depth of Fe_3_O_4_ NPs and F) Hu within MRSA biofilm under different therapeutic conditions calculated from (D) (*n* = 10, means ± SD). G) 3D CLSM images of MRSA biofilm stained by diaminofluorescein‐FM diacetate (DAF‐FM DA, NO probe) after various treatments with the addition of H_2_O_2_ (100 µM). Scale bar is 200 µm. H) Fluorescence intensity (*n* = 5, means ± SD) and I) penetration depth of NO in biofilms after various treatments calculated from (G) (*n* = 10, means ± SD).

Low‐intensity US can stimulate Fe_3_O_4_ NPs‐shelled MBs in a stable oscillation manner. When the US intensity is higher than the cavitation threshold of MBs, the MBs can undergo inertial cavitation, and collapse by cavitation effect for rapid drug release, which may damage the surrounding tissues.^[^ ^16^, ^21^ ^]^ Compared with the high‐intensity US‐triggered inertial cavitation of MBs, the low‐intensity US can stimulate stable oscillation of MBs for gradual drug release, and such an “exocytosis”‐like manner of MBs may not only reduce the risk of tissue injury in cavitation but also exert impact stress on released agents for crossing the biofilm barrier.^[^ ^16^, ^22^ ^]^ Therefore, the low‐intensity US (according to Figure 2J,K, 0.5 W cm^−2^) was used in this work. As illustrated in **Figure** **3**A, MRSA biofilms treated by erythrocyte membrane fragments encapsulated Fe_3_O_4_ NPs (EFe) with Hu and US stimulation or EMB‐Hu retained the contact biofilm structure, while those treated by EMB‐Hu with US stimulation showed distinct disruption of biofilm structure. In addition, the thickness and biomass of MRSA biofilms treated by EMB‐Hu with US stimulation were decreased compared to those treated by EFe with Hu under US stimulation or EMB‐Hu (Figure 3B,C), indicating that the US‐stimulated stable oscillation of EMB‐Hu can disrupt the MRSA biofilm.

The penetration of Fe_3_O_4_ NPs and Hu in MRSA biofilms was then evaluated in vitro. The transwell assay indicated that US stimulation can enhance the penetration of Hu in MRSA biofilms after treatment with EMB‐Hu (Figure S17A, Supporting Information). After being treated by EMB‐Hu with US stimulation, the penetration depths of Fe_3_O_4_ NPs and Hu were ≈38.2 and 40.1 µm, respectively, which were much higher than those in the EFe + Hu + US and EMB‐Hu groups (Figure 3D–F), and the amount of penetrated Hu in EMB‐Hu + US group was higher than that in the EMB‐Hu and Hu + US groups (Figure S17B, Supporting Information), indicating that EMB‐Hu with US stimulation can enhance the penetration of Fe_3_O_4_ NPs and Hu in MRSA biofilms. The generation of NO and ROS within MRSA biofilms was further investigated by staining with diaminofluorescein‐FM diacetate (DAF‐FM DA, NO probe) and 2′,7′‐dichlorofluorescin diacetate (DCFH‐DA, ROS probe). As shown in Figure 3G–I and Figure S18A,B, Supporting Information, the obvious fluorescence signal of NO and ROS can be observed in EMB‐Hu with US stimulation‐treated biofilms, while EMB‐Hu treated biofilm showed a limited fluorescence signal of NO and ROS, indicating US‐stimulated NO and ROS generation capability of EMB‐Hu with the presence of H_2_O_2_. Importantly, the penetration depths of NO and ROS within biofilms after being treated by EMB‐Hu with US stimulation were 24.5 and 25.2 µm, respectively, which were much higher than EFe + Hu + US group (9.4 µm for NO and 18.7 µm for ROS, Figure S18C, Supporting Information). These results demonstrated that the EMB‐Hu under US stimulation can disrupt the MRSA biofilm structure, enhance the penetration of Fe_3_O_4_ NPs and Hu, and further improve the diffusion of NO and ROS within biofilms.

### 2.3 Treatment of MRSA Biofilms In Vitro

**Figure 4:**
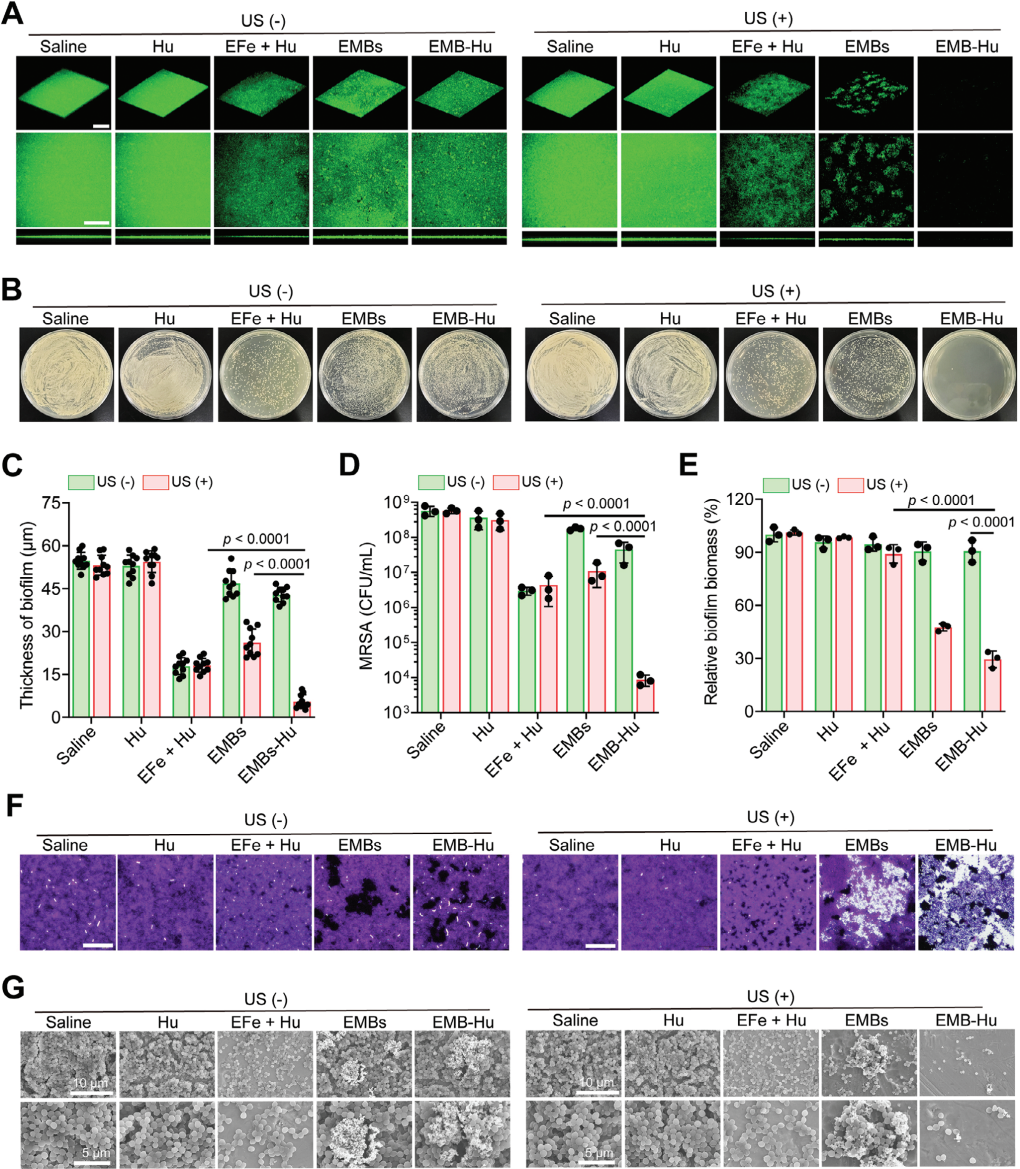
Therapeutic efficacy of MRSA biofilm after various treatments in vitro. A) 3D CLSM images of biofilm stained by Calcein‐AM with the presence of H_2_O_2_ (Fe_3_O_4_ NPs: 500 µg mL^−1^; Hu: 150 µg mL^−1^; H_2_O_2_: 100 µM; US: 1 MHz, 0.5 W cm^−2^, 50% amplitude, 10 min). Scale bar is 200 µm. B) Photographs of MRSA colonies after various treatments with the presence of H_2_O _2_ . C) Thickness of MRSA biofilm in different groups calculated from (A) (*n* = 10, means ± SD). D) the number of live bacteria within MRSA biofilms in different groups with the presence of H_2_O_2_ (*n* = 3, means ± SD). E) Relative biofilm biomass and F) microscope images of biofilm stained by crystal violet with the presence of H_2_O_2_ in different groups (*n* = 3, means ± SD). Scale bar is 200 µm. G) SEM images of MRSA biofilms with the presence of H_2_O_2_ in different groups.

The therapeutic efficiency of EMB‐Hu for biofilms was then determined in vitro. As illustrated in Figure S19, Supporting Information, EMB‐Hu had a limited anti‐bacterial effect on MRSA biofilms. Under US stimulation, EMB‐Hu also showed no anti‐bacterial effect on MRSA biofilms, indicating that only US‐stimulated disruption of biofilms by EMB‐Hu can hard inactive bacteria within biofilms. In the presence of H_2_O_2_, EMB‐Hu with US stimulation can inactive bacteria (4.5 log units, 99.997%) within MRSA biofilms, while EMB‐Hu with H_2_O_2_ can only inactivate 85% (0.73 log units) of bacteria within biofilms, indicating that US‐stimulated biofilm disruption and ROS/NO generation by EMB‐Hu can efficiently kill bacteria within biofilms. The comparison of different agents for the treatment of MRSA biofilms was further tested. All experimental groups were added with 100 µM H_2_O_2_ to mimic the biofilm‐infected tissue with the overexpressed H_2_O_2_. As shown in **Figure** **4**A,C, Hu showed a limited bacterial inactivation effect. After being treated by EFe + Hu with H_2_O_2_, the thickness of the MRSA biofilm was reduced to ≈19.2 µm, while that in the EMB + US + H_2_O_2_ group was ≈23.4 µm. The biofilm in the EMB‐Hu + US + H_2_O_2_ group remained at the lowest thickness of ≈5.7 µm. Besides, the biofilm in the EMB‐Hu + US + H_2_O_2_ group exhibited the highest reduction of bacteria, with ≈4.6 log units (99.998%), while that in the EFe + Hu + US + H_2_O_2_ and EMBs + US + H_2_O_2_ groups was only ≈1.8 log units (98.5%) and 1.6 log units (96.7%), indicating that US‐stimulated biofilm disruption by EMB‐Hu can efficiently enhance the antibacterial effect of catalytically generated ROS and NO (Figure 4B,D). After being treated with EFe + Hu with H_2_O_2_, MRSA biofilms remained intact structure, indicating that only ROS and NO generation can hardly decompose the biofilm structure. In contrast, the structure of MRSA biofilms was obviously decomposed (70.3%) after being treated with EMB‐Hu + US + H_2_O_2_ (Figure 4E,F). The EMB‐Hu can also inactivate ≈4.0 log units (99.99%) of bacterial inactivation and decompose 55.6% of biofilm biomass under US stimulation with the presence of H_2_O_2_ for the MRSA biofilm growth in titanium plate (Figure S20, Supporting Information). The SME images indicated that the MRSA biofilm has been almost eliminated after being treated with EMB‐Hu + US + H_2_O_2_ (Figure 4G). All results indicated that US‐stimulated disruption of biofilm can efficiently promote the biofilm elimination efficiency of catalytically generated ROS and NO by EMB‐Hu.

**Figure 5:**
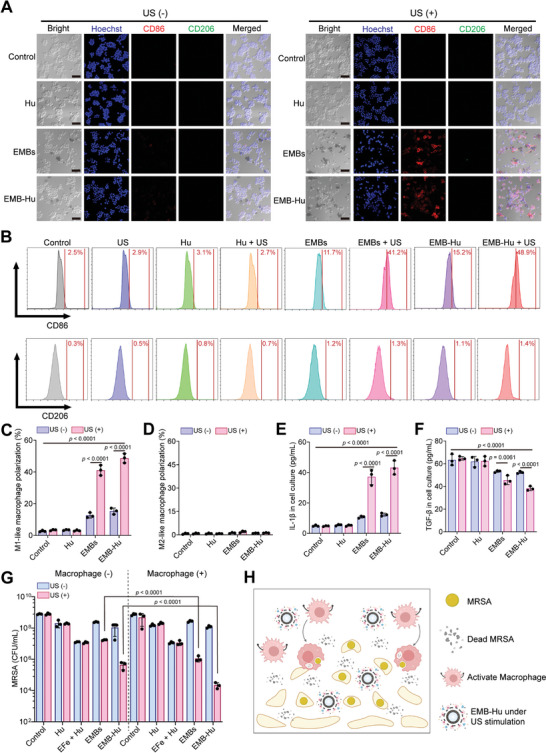
US stimulated macrophage activation by EMB‐Hu in vitro. A) CLSM images of RAW 264.7 cells stained with Hoechst (blue), PE‐labeled CD86 (red), and APC‐labeled CD206 (green) in different groups (Fe_3_O_4_ NPs: 500 µg mL^−1^; Hu: 150 µg mL^−1^; US: 1 MHz, 0.5 W cm^−2^, 50% amplitude, 10 min) for 24 h. Scale bar is 50 µm. B) Flow cytometry analysis of CD86^+^ or CD206^+^ RAW264.7 cells in different groups. C) Polarization of M1‐like macrophages (CD86^+^) and D) M2‐like macrophages (CD206^+^) in different groups (*n* = 3, means ± SD). E) Secretion of IL‐1β and F) TGF‐β in RAW264.7 cells in different groups (*n* = 3, means ± SD). G) Colony number of live bacteria in MRSA biofilms in different groups (containing H_2_O_2_, 100 µM) with or without the addition of RAW264.7 cells (10^5^ per well) (*n* = 3, means ± SD). H) US‐stimulated biofilm disruption and macrophage activation by EMB‐Hu for efficient biofilm elimination.

The formation of MRSA biofilms can not only inhibit the phagocytosis effects of macrophages but also facilitate anti‐inflammatory M2 macrophage polarization to restrict the bacterial clearance effect of macrophages.^[^ ^3^, ^4^, ^8^ ^]^ Previous works indicated that Fe_3_O_4_ NPs can promote M1‐like macrophage polarization to enhance pro‐inflammatory responses for the benefit of bacterial inactivation.^[^ ^23^ ^]^ Therefore, we assume that the US‐stimulated “exocytosis” by EMB‐Hu‐induced disruption of biofilm can enhance the phagocytosis effect of immune cells, and the released Fe_3_O_4_ NPs can also act as “signaling molecules” for activating macrophages to eliminate the disrupted biofilms. First, the mouse leukemia cells of monocyte‐macrophage (RAW 264.7 cells) were used to determine the polarization effect of EMB‐Hu on macrophages in vitro. After incubation with EMBs and EMB‐Hu for 24 h, the polarization of M1‐like macrophages was increased to ≈11.2% and 15.2%, respectively (**Figure** **5**A–D). Under US stimulation, EMBs and EMB‐Hu induced ≈41.2% and 48.9% M1‐like macrophage polarization, respectively, which were much higher than EMBs and EMB‐Hu groups, suggesting that US‐stimulated release of Fe_3_O_4_ NPs by EMB‐Hu can activate the polarization of M1‐like macrophages. The RAW 264.7 cells in all groups showed limited polarization of M2‐like macrophages. In addition, an obvious increase in M1‐like macrophage‐related cytokines (interleukin‐1β, IL‐1β) and a decrease in M2‐like macrophage‐related cytokines (transforming growth factor‐β, TGF‐β) were observed in the EMBs + US and EMB‐Hu + US groups (Figure 5E,F), which further confirms the US‐stimulated macrophage activation effect of EMBs‐Hu.

The therapeutic effects of EMB‐Hu for MRSA biofilms with the addition of macrophages were further evaluated. As illustrated in Figure 5G, the bacterial number within biofilms in the EMBs + US + macrophage group was reduced by ≈2.2 log units (99.4%), which was higher than that in the EMBs + US group (1.3 log units, 94%), while the number of bacteria within MRSA biofilms in the EFe + Hu + macrophage group showed limited change compared with that in the EFe + Hu group. The bacterial inactivation efficiency in the EMB‐Hu + US + macrophage group with ≈4.9 log units’ reduction of MRSA (99.999%) was greatly higher than that in the EMB‐Hu + US group (3.4 log units’ reduction, 99.95%), which can be assigned to the enhanced phagocytosis of activated macrophages for the disrupted biofilms (Figure S21, Supporting Information). These results indicated that US‐stimulated biofilm disruption and macrophage activation by EMB‐Hu can enhance the bactericidal effect of macrophages on MRSA biofilms (Figure 5H).

### 2.4 US‐Stimulated Agent Penetration and Immune Activation by EMB‐Hu In Vivo

**Figure 6:**
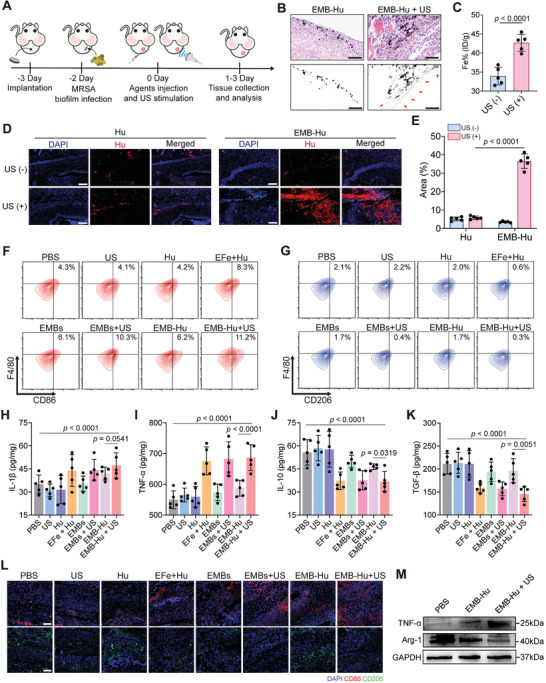
US‐stimulated agent penetration and immune activation by EMB‐Hu. A) Scheme of the therapeutic strategies for MRSA biofilm‐induced implant infection in a mouse model. B) Microscope images of the slice for the infected tissue stained by H&E after various treatments. Red arrows indicate the penetration of Fe_3_O_4_ NPs. Scale bar is 200 µm. C) Accumulation of Fe in infected tissues after various treatments (*n* = 3, means ± SD). D) Fluorescence images of the slice for the infected tissue after various treatments (blue: DAPI, red: Rh‐Hu) and E) Rh‐Hu positive area calculated from (D) (*n* = 5, means ± SD). Scale bar is 200 µm. F) Flow cytometry analysis of M1‐like macrophages (CD11b^+^F4/80^+^CD86^+^) and G) M2‐like macrophages (CD11b^+^F4/80^+^CD206^+^) infiltrated in infected tissues after various treatments for 3 days. H) Secretion of IL‐1β, I) TNF‐α, J) IL‐10, and K) TGF‐β in tissues after various treatments for 3 days by ELISA (*n* = 5, means ± SD). L) Immunofluorescence images of the slice for the infected tissue stained by DAPI (blue), CD86 (red), and CD206 (green) after various treatments for 3 days. Scale bar is 100 µm. M) Expression of TNF‐α and Arg‐1 in infected tissues after various treatments tested by western blotting.

To determine the therapeutic effect of EMB‐Hu in vivo, MRSA biofilm‐induced implant infection was fabricated in a mouse model. First, the titanium sheet was implanted in the thigh of mice for 1 d, and then infected with MRSA for 2 d to form the MRSA biofilm‐indued implant infection in a mouse model (**Figure** **6**A).^[^ ^2^, ^4^ ^]^ As shown in Figure 6B,C, the EMB‐Hu with US stimulation can promote the penetration and accumulation of Fe_3_O_4_ NPs in biofilm‐infected tissues. Compared with only Hu‐injected mice with US stimulation, the penetration of Hu in EMB‐Hu infected mice with US stimulation was obviously increased (Figure 6D,E), suggesting the US‐stimulated Fe_3_O_4_ NPs and Hu penetration by EMB‐Hu in the MRSA biofilm infected tissue. In addition, the US‐stimulated Fe_3_O_4_ NPs and Hu penetration by EMB‐Hu can further promote the generation and diffusion of ROS and NO in biofilm‐infected sites (Figure S22, Supporting Information).

The immunoregulatory effects in vivo of EMB‐Hu in MRSA biofilm‐induced implant infection were further evaluated. As illustrated in Figures S23 and S24, Supporting Information, the flow cytometry results indicated that the infiltration of macrophages (CD11b^+^F4/80^+^) in EMBs + US and EMB‐Hu + US groups were ≈2.5% and 2.6%, respectively, which was higher than that in the only MBs treated groups (EMBs and EMB‐Hu) and the EFe + Hu group (≈1.8%). In addition, the polarizations of M1‐like macrophages (CD11b^+^F4/80^+^CD86^+^) in EMBs + US and EMB‐Hu + US groups were obviously increased to 10.3% and 11.2, respectively, while that in PBS and EMB‐Hu groups was only 4.3% and 6.2%, respectively. In contrast, the polarization of M2‐like macrophage (CD11b^+^F4/80^+^CD206^+^) in EMBs + US and EMB‐Hu + US groups were obviously decreased compared with that in the PBS group (Figure 6F,G, and Figure S24C, Supporting Information). The results of the enzyme‐linked immunosorbent assay (ELISA) indicated a distinct increase in M1‐like macrophage‐related cell cytokines (IL‐1β and tumor necrosis factor‐α, TNF‐α) and a decrease in M2‐like macrophage‐related cell cytokine secretions (IL‐10 and TGF‐β) in EMBs + US and EMB‐Hu + US groups comparing with EMB‐Hu and PBS groups (Figure 6H–K). In addition, the immunofluorescent images and the results of the western blotting assay further indicated enhanced expression of CD86 and TNF‐α and decreased expression of CD206 and arginase‐1 (Arg‐1) in the EMB‐Hu + US group compared with the PBS and EMB‐Hu groups (Figure 6L,M). These results suggested the US‐stimulated biofilm disruption and Fe_3_O_4_ NPs penetration can enhance the infiltration of macrophages in infected sites, and facilitate the polarization of M1‐like macrophages.

### 2.5 Treatment of Biofilm‐Associated Implant Infection In Vivo

**Figure 7:**
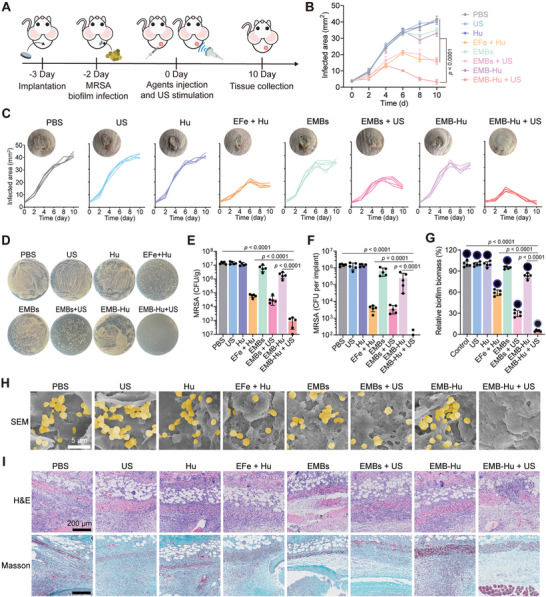
Treatment of MRSA biofilm‐associated implant infection in vivo. A) Schematic illustration of the strategies for the treatment of biofilm‐induced implant infection in a mouse model. B) Average and C) individual infection area growth curves of MRSA biofilm‐infected mice in different groups (*n* = 5, means ± SD). Inserts: representative photos of infected tissue after indicated treatments for 10 days. D) Pictures of MRSA colonies in plate and E) corresponding colony number of live MRSA in infected tissues in different groups for 10 days (*n* = 5, means ± SD). F) Colony number of live bacteria in implants collected from infected tissues in different groups for 10 days (*n* = 5, means ± SD). G) Relative biofilm biomass of MRSA biofilm in implants collected from infected tissues in different groups for 10 days (*n* = 5, means ± SD). Inserts: Picture of crystal violet‐stained implants collected from infected tissues. H) SEM images of implants collected from infected tissues in different groups for 10 days. The pseudo‐colored yellow indicates the location of MRSA. I) Microscopy images of the slice stained by H&E and Masson for the infected tissues in different groups for 10 days.

For the treatment of MRSA biofilm‐induced implant infection in a mouse model, different agents were subcutaneously injected into infected tissue, stimulated with the US in US‐treated groups, and the healing of infected tissues was observed (**Figure** **7**A). The infected area in PBS, Hu, EMBs, and EMB‐Hu groups gradually increased after treatments for 10 days, while that in the EMB‐Hu + US group increased post 4 days of treatment, and then decreased quickly (Figure 7B,C). They showed limited infected areas in the EMB‐Hu + US group post‐10 days of treatment, while those in other groups still remained infected areas. On the other hand, the live bacteria were reduced by ≈4.2 log units (99.994%) in infected tissue and 4.1 log units (99.993%) in the implant for the EMB‐Hu + US group compared with the PBS group, which were much higher than EFe + Hu and EMB‐Hu groups (Figure 7D–F). The images of the crystal violet‐stained implant and SEM images of the implant further indicated the almost disappearance of MRSA biofilm in the implant for the EMB‐Hu + US group, while that in other groups still retained biofilms on the surface of the implant (Figure 7G,H). Besides, there was no bacteria dissemination into the bloodstream and no obvious inflammatory response in the major organs (heart, liver, spleen, lung, and kidney) from infected mice after treatment with EMB‐Hu with US stimulation (Figure S25, Supporting Information). These results indicated that US‐stimulated biofilm disruption, anti‐bacterial species penetration, and immune activation by EMB‐Hu can efficiently eliminate MRSA biofilms in implants. The images of H&E and Masson staining slices for the infected tissue showed slight infiltration of inflammatory cells, fibroblast proliferation, and neovascularization in the EMB‐Hu + US group, while the massive infiltration of inflammatory cells and impaired hypodermis structure were displayed in other groups (Figure 7I), suggesting the good therapeutic‐effects of EMB‐Hu under US stimulation for MRSA biofilm‐induced implant infections. Importantly, the serum biochemical parameters and routine blood examination of mice intravenously injected with EMB‐Hu showed limited differences compared with healthy mice (Figure S26A–H, Supporting Information). H&E‐stained sections of major organs from mice injected with EMB‐Hu also showed no observable damage or inflammatory responses (Figure S26I, Supporting Information), suggesting the low toxicity of EMB‐Hu in vivo.

## 3 Conclusions

In this work, we fabricated cell‐like MBs by facile assimilation of erythrocyte membrane fragments with Fe_3_O_4_ NPs fabricated MBs loaded with Hu (EMB‐Hu) to treat MRSA biofilm‐associated implant infection. Natural cellular exocytosis is a process for actively transporting agents from within a cell to the exterior of the cell by consuming energy, but the released agents are without the mechanical stress overcoming the physiological barrier. Owing to the specific acoustic properties of MBs, EMB‐Hu can undergo volumetric oscillations under low‐intensity US stimulation, which induces the acceleration of the bubble wall to impose substantial forces on nearby surfaces, and further enables agent inside the MBs to be released from the exterior of MBs. Meanwhile, the momentum generated from oscillating MBs can induce the interfacial friction of MBs with the surrounding liquid to produce a localized generation of microstreaming (Figure 2I–K), and enable released agents with mechanical force to overcome the physiological barrier. As the natural exocytosis process of immune cells is insufficient in inducing the anti‐bacterial species to overcome the biofilm barrier, resulting in inhibited biofilm inactivation, such US‐stimulated “exocytosis” by EMB‐Hu has been shown to physically disrupt MRSA biofilms (Figure 3A–C), facilitate agent penetration within biofilms (Figure 3D–F), and ultimately enhance the diffusion of catalytically generated anti‐bacterial species (ROS and NO) for improved bacterial inactivation (Figures 3G–I and 4, and Figure S18, Supporting Information). On the other hand, the biofilm can also restrict the pathogen clearance effect of immune cells through encapsulation of EPS to inhibit phagocytosis and secrete toxicity factors to frustrate inflammatory activation of immune cells. The US‐stimulated biofilm disruption by EMB‐Hu may enhance the phagocytosis of immune cells for bacterial inactivation, but the frustrated immune cells during biofilm infection can hardly be recruited to the infection site for rapid biofilm elimination, which leads to the risk of biofilm reformation. Herein, the Fe_3_O_4_ NPs released from EMB‐Hu under US stimulation can act as a “signaling molecule” to activate the pro‐inflammatory macrophage polarization (Figure 5A–F), which not only promotes the infiltration of macrophages in the infected tissue but also increases the phagocytosis of macrophages for efficient elimination of disrupted biofilm both in vitro (99.997%) and in vivo (99.994%) (Figures 5, 6, and 7D–H, and Figures S21 and S24, Supporting Information). Therefore, our demonstrated cell‐like EMB‐Hu with US‐stimulated “exocytosis” capability achieved biofilm disruption, enhanced antibacterial species penetration, and signaling‐elevated inflammatory immune activation for efficient treatment of MRSA biofilm‐induced implant infection in a mouse model. Although the EMB‐Hu with US stimulation showed promising bacterial biofilm elimination efficiency, incomplete biofilm elimination leaves a risk for the recurrence of biofilms. On the one hand, the remained bacteria within the biofilm can further proliferate in the infected tissue and further induce the regrowth of biofilms. On the other hand, the residual biofilm substrate on the substrate makes it easier for planktonic bacteria to adhere to the surface of the substrate, further causing a secondary infection. Therefore, how to prevent the regrowth of biofilm in the infected site is another challenge that needs to be addressed in further work.

Nonetheless, there still exist opportunities for further improvement of the EMB‐Hu. In contrast to the natural exocytosis process involving transmembrane transport, the US‐stimulated “exocytosis” by EMB‐Hu merely emulates the process of cell secretion of substances, thereby inducing the continuous release of components from MBs and eventually leads to the collapse of MBs, which is unsuitable for long‐term disease treatments. Additionally, the Fe_3_O_4_ NPs as components of MBs, are difficult to degrade in vivo, posing potential risks for future clinical translation. Although many gaps remain between such prepared cell‐like constructs and natural immune cells, the design principle described here will provide a framework for developing next‐generation therapeutic cell‐like constructs with the aim of improving cell‐function deficiencies for a long period of time.

## 4 Experimental Section

### Materials

Hydroxyurea (Hu, ≥ 98%), sodium dodecyl sulfate (SDS, ≥ 98.5%), 3,3′,5,5′‐tetramethylbenzidine (TMB, ≥ 99%), 2′,7′‐dichlorofluorescin diacetate (DCFH‐DA, ≥ 97%), and diaminofluorescein‐FM diacetate (DAF‐FM DA, ≥ 98%) were obtained from Sigma‐Aldrich. Ferric oxide nanoparticles (Fe_3_O_4_ NPs, ≈100 nm) were purchased from Alfa Aesar (USA). Rhodamine B‐labeled Hu (Rh‐Hu) was purchased from Shiyanjia Lab (China). The 1,1′‐dioctadecyl‐3,3,3′,3′‐tetramethylindocarbocyanine perchlorate (Dil) was brought from ThermoFisher Scientific. Glutaric dialdehyde (25%) and hydrogen peroxide (H_2_O_2_, 30 wt %) solutions were obtained from Sinopharm Chemical Reagent Co., Ltd. (China).

### Preparation of Erythrocyte Membrane Fragments‐Encapsulated Hu‐Loaded Microbubbles

The drug‐loaded MBs were prepared according to the previous works.^[^ ^17^ ^]^ Briefly, the Fe_3_O_4_ NP aqueous dispersions (10 mg ml^−1^, 400 µL), SDS solution (10 mM, 150 µL), and Hu (5 mg mL^−1^, 150 µL) solution were mixed and homogenized at 20 000 rpm for 3 min. Then, the mixture was stored for 24 h for close packing of Fe_3_O_4_ NPs, separated by the magnet, and washed with H_2_O three times to form Hu‐loaded microbubbles (MB‐Hu).

For the collection of erythrocyte membrane fragments, an aqueous suspension of erythrocytes (10 ml, 40% v/v) collected from Balb/c mice was first centrifuged at 2000 r.p.m. for 10 min (4 °C condition), washed with DPBS (Dulbecco's phosphate‐buffered saline) three times, and then dispersed in 25% DPBS solution in an ice bath for 1 h to obtain the lysed erythrocytes. The lysed erythrocyte solutions were centrifuged at 4000 r.p.m. for 20 min (4 °C condition) to collect the sediment and then washed with DPBS two times to obtain erythrocyte membrane fragments. The erythrocyte membrane fragments were finally freeze‐dried and stored at −20 °C.

To prepare erythrocyte membrane fragments‐encapsulated MB‐Hu (EMB‐Hu), freeze‐dried erythrocyte membrane fragments were dispersed in H_2_O (0.1 mg mL^−1^, 1 mL), added to MB‐Hu (Fe_3_O_4_ NPs: 1 mg; Hu: 0.3 mg), and then incubated at 4 °C for 12 h. The MBs were finally separated by the magnet and washed with H_2_O three times to obtain erythrocyte membrane‐encapsulated MB‐Hu (EMB‐Hu). The contents of Fe_3_O_4_ NPs and Hu in EMB‐Hu were measured by inductively coupled plasma‐optical emission spectrometry (ICP‐OES, PerkinElmer, USA) and spectrophotometry (Shimadzu UV‐3600, Japan), respectively. The content of protein in EMB‐Hu was measured by a BCA protein quantification kit (Beyotime Biotechnology).

### US‐Stimulated Agents Released by EMB‐Hu

EMB‐Hu (Fe_3_O_4_ NPs: 250 µg; Hu: 75 µg) were dispersed in H_2_O and stimulated with US (1 MHz, 50% amplitude) with different frequencies (0.2, 0.5, 1.0, and 2.0 W cm^−2^) for different times. After removing EMB‐Hu by using the magnet, the released Fe_3_O_4_ NPs and Hu in solution were quantified by using ICP‐OES and spectrophotometry, respectively.

### Detection of ROS and NO Generation In Vitro

The MRSA biofilms were grown in the confocal culture dish and then added to EFe + Hu (Fe_3_O_4_ NPs: 0.5 mg mL^−1^; Hu: 0.15 mg mL^−1^, 200 µL) and EMB‐Hu (Fe_3_O_4_ NPs: 0.5 mg mL^−1^; Hu: 0.15 mg mL^−1^, 200 µL) with the addition of H_2_O_2_ (100 µM), stimulated with US (1 MHz, 0.5 W cm^−2^, 50% amplitude, 10 min), and then incubated for 1 h. After staining with DAF‐FM DA (10 µM, NO probe) or DCFH‐DA (10 µM, ROS probe), and Hoechst for 30 min, the MRSA biofilms were observed by using a confocal laser scanning microscope (CLSM, Olympus IX81).

### Treatment of MRSA Biofilms In Vitro

The MRSA biofilm was incubated with PBS, Hu, EFe + Hu, EMBs, and EMB‐Hu (Fe_3_O_4_ NPs: 0.5 mg mL^−1^; Hu: 0.15 mg mL^−1^, 200 µL) with the addition of H_2_O_2_ (100 µM), stimulated with US (1 MHz, 0.5 W cm^−2^, 50% amplitude, 10 min), and then incubated for 12 h. The MRSA biofilms after different treatments were harvested, slightly washed with saline three times to remove the materials, then dispersed in saline under ultrasonic cleaner (108 W, 10% amplitude) for 5 min to completely disperse the biofilm, and finally quantified the number of live MRSA within biofilm by using the standard plate counting method. In detail, the MRSA dispersions were diluted 10 times serially. The 100 µL of diluted MRSA dispersion was plated into the LB agarose plate and incubated these plates upside down at 37 °C for 24 h. Select the plates that have between 30 and 300 colonies produced, and finally multiply the number of colonies counted by the dilution factor to calculate the number of live MRSA in the original solution. For fluorescence imaging, the treated MRSA biofilms were stained with Calcein‐AM (KeyGen BioTech) and observed using CLSM (Olympus IX81). For the quantification of biomass for the biofilm, the treated MRSA biofilms were washed with saline three times, and fixed with formalin for 30 min. After natural drying, the fixed biofilms were stained with crystal violet solution (2%) for 20 min, washed with H_2_O three times, and finally decolorized with alcohol for 1 h. The absorbance at 570 nm of all samples was measured by using a microplate reader (PowerWave XS2, BioTek) to calculate the relative biofilm biomass of treated biofilms. To evaluate the an‐biofilm effects of different agents with the addition of macrophages, the MARS biofilm after various treatments was further incubated with RAW 264.1 (10^5^ cells per well) in DMEM (containing 10% FBS) under a 5% CO_2_ atmosphere at 37 °C conditions for 24 h. All samples were collected, dispersed under ultrasonic cleaning, centrifuged at 12 000 r.p.m. for 5 min, and finally calculated the live MRSA by using the standard plate counting method. Each group conducted three biological replicates as parallel experiments.

### Animals

Female Balb/c mice (∼20 g, 6–8 weeks old) were purchased from Shanghai SLAC Laboratory Animal Co., Ltd. All animal procedures were performed in accordance with the Guidelines for the Care and Use of Laboratory Animals of Nanjing University and approved by the Animal Ethics Committee of Nanjing University. All mice were fed at 25 ± 3 °C, 60–70% humidity, and 12 h light/dark cycle conditions.

### Treatment of MRSA Biofilm‐Induced Implant Infection In Vivo

To construct the MRSA biofilm‐induced implant infection in a mouse model, the Balb/c mice were anesthetized, shaved, and disinfected. Sterile titanium plates (diameter, 6 mm) were inserted into the right thigh of mice and grown for 1 day. Then, 50 µL of MRSA (10^8^ CFU per mL) dispersed in LB (containing 1% glucose) were subcutaneously injected into the implant and infected for 2 days. The infected mice were randomly allocated into eight groups, subcutaneously injected with 50 µL of different agents (PBS; Hu: 0.3 mg mL^−1^; EFe + Hu: 1 mg mL^−1^ Fe_3_O_4_ NPs and 0.3 mg mL^−1^ Hu; EMBs: 1 mg mL^−1^ Fe_3_O_4_ NPs; EMB‐Hu: 1 mg mL^−1^ Fe_3_O_4_ NPs and 0.3 mg mL^−1^ Hu) in infected sites, and stimulated with US (1 MHz, 0.5 W cm^−2^, 50% amplitude, 10 min) in the US‐treated groups. Each group conducted five biological replicates as parallel experiments. The infected sites of each mouse were monitored post‐treatment, and the infected area was calculated according to the formula: Area = (width/2 × length/2) × π. After treatments for 10 days, all implants and surrounding infected tissues were collected to analyze live bacteria by using the standard plate counting method. For histological examination, the infected tissues were fixed with formalin for 24 h, embedded in paraffin, and sliced for hematoxylin and eosin (H&E), Masson's trichrome, and Giemsa staining.

### Polarization of Macrophages In Vivo

The infected tissues were collected from mice after various treatments for 3 days. The infected tissues were clipped into small pieces, digested by using type I collagenase (2 mg mL^−1^, Gibco) and deoxyribonuclease (100 µg mL^−1^, Sigma‐Aldrich) for 30 min at 37 °C, and filtered through 50 µm filters to collect single‐cell suspensions. After lysing RBCs by using ACK lysis buffer (Gibco) for 5 min, the single‐cell suspensions were first incubated with anti‐mouse CD16/32 antibody (BioLegend, catalog no. 101301, clone 93) for 10 min to block Fc receptors, stained with Zombie Violet (BioLegend, catalog no. 423113) to isolate live cells, and finally stained with FITC anti‐mouse CD11b (BioLegend, catalog no. 101205, clone M1/70), PerCP‐Cy5.5 anti‐mouse F4/80 (BioLegend, catalog no. 123125, clone BM8), PE anti‐mouse CD86 (BioLegend, catalog no. 159203, clone A17199A), and APC anti‐mouse CD206 (BioLegend, catalog no. 141707, clone C068C2) antibodies for 30 min following the manufacturer's instructions. The stained cells were then measured by using an SA3800 flow cytometry (SONY, Japan) and analyzed with FlowJo software. The levels of TNF‐α (Abcam, catalog no. ab208348), IL‐10 (Abcam, catalog no. ab255729), IL‐1β (Abcam, catalog no. ab197742), and TGF‐β (Abcam, catalog no. ab119557) were measured by enzyme‐linked immunosorbent assay kits according to the manufacturer's instructions.

For immunofluorescence staining, the infected tissues were snap‐frozen by using liquid nitrogen, cut by cryotome, and finally stained with primary antibody (anti‐F4/80 antibody, Abcam, catalog no. ab6640; anti‐86 antibody, Abcam, catalog no. ab119857; anti‐206 antibody, Abcam, catalog no. ab64693), fluorescently labeled secondary antibody, and 4′,6‐diamidino‐2‐phenylindole (DAPI) following the manufacturer's instruction. All slices were imaged using a CLSM (Olympus IX81). For the Western‐blotting assay, the primary antibodies (TNF‐α antibody, Cell Signaling Technology, Cat. #11948, Arginase‐1 antibody, Cell Signaling Technology, Cat. #93668, GAPDH antibody, Cell Signaling Technology, Cat. #5174) were used in this work. All antibodies were diluted 200 times before use.

### Statistical Analysis

The data were expressed as the mean ± SD. Inter‐group and intra‐group comparison analyses in each experiment were calculated through one‐ or two‐way ANOVA with a Tukey's post‐hoc test by using GraphPad Prism. *p*‐values < 0.05 were considered statistically significant.

## Conflict of Interest

The authors declare no conflict of interest.

## Supporting information

Supporting Information

## Data Availability

The data that support the findings of this study are available from the corresponding author upon reasonable request.
