# Supplementary material for: Ultrasound‐Stimulated “Exocytosis” by Cell‐Like Microbubbles Enhances Antibacterial Species Penetration and Immune Activation Against Implant Infection
Source: Adv Sci (Weinh). 2023 Dec 18;11(10):2307048. doi: 10.1002/advs.202307048 (PMC10933665; doi:10.1002/advs.202307048)
Supplement: Supplementary file 1 — Supporting Information [file ADVS-11-2307048-s001.pdf]

ADVANCED SCIENCE
Open Access

Supporting Information

for *Adv. Sci.*, DOI 10.1002/advs.202307048

Ultrasound-Stimulated “Exocytosis” by Cell-Like Microbubbles Enhances Antibacterial Species Penetration and Immune Activation Against Implant Infection

Weijun Xiu, Xiaoye Li, Qiang Li, Meng Ding, Yu Zhang, Ling Wan, Siyu Wang, Yu Gao, Yongbin Mou, Lianhui Wang* and Heng Dong**

Supporting Information

Ultrasound-stimulated "exocytosis" by cell-like microbubbles enhances antibacterial species penetration and immune activation against implant infection

Weijun Xiu[#], Xiaoye Li[#], Qiang Li, Meng Ding, Yu Zhang, Ling Wan, Siyu Wang, Yu Gao, Yongbin Mou, and Lianhui Wang*, and Heng Dong**

W.X., L.X., Q.L., M.D., Y.Z., Prof. Y.M., Prof. H.D.
Nanjing Stomatological Hospital, Affiliated Hospital of Medical School, Nanjing University
30 Zhongyang Road, Nanjing, 210008 Jiangsu, P.R. China
E-mail: Y.M. yongbinmou@nju.edu.cn H.D. dongheng90@smail.nju.edu.cn

W.X., S.W., Prof. Y.G., Prof. L.W.
Key Laboratory for Organic Electronics and Information Displays
Jiangsu Key Laboratory for Biosensors, Institute of Advanced Materials
Jiangsu National Synergetic Innovation Centre for Advanced Materials
Nanjing University of Posts and Telecommunications
9 Wenyuan Road, Nanjing, 210023 Jiangsu, P.R. China
E-mail: L.W. *iamlhwang@njupt.edu.cn*

Supplementary Methods

Cell and bacteria culture: Human oral keratinocytes (HOK) and RAW 264.7 macrophage cells (obtained from KeyGen BioTech) were cultured in Dulbecco's modified Eagle's medium (DMEM, KeyGEN BioTECH) containing 10% fetal bovine serum (FBS, Gibco), 1% penicillin, and 1% streptomycin in a 5% CO_2 atmosphere at 37°C conditions. The methicillin-resistant *Staphylococcus aureus* (MRSA, ATCC43300) was obtained from the American Type Culture Collection, and grown in Luria-Bertani (LB) medium for 10 h to collect the planktonic MRSA. For determining the concentration of MRSA dispersion, the absorbance of MRSA dispersed in saline at 600 nm (OD_{600}) was measured by using the microplate reader (PowerWave XS2, BioTek). The OD_{600} = 0.1 indicates the concentration of MRSA dispersion is 10^7 CFU/mL. For the formation of MRSA biofilms, the planktonic MRSA (10^7 CFU/mL) was incubated in LB medium (containing 1% glucose, 10^7 CFU/mL) at 37 °C for 2 d.

Peroxidase-like catalytic activity: The peroxidase-like catalytic activity of different agents was evaluated by using 3,3',5,5'-Tetramethylbenzidine (TMB) as a substrate. The US-treated agents were prepared by stimulation with US (1 MHz, 0.5 W/cm^2, 50% amplitude) for 10 min. The various agents in 0.2 M NaAc-Hac buffer (pH 3.6; 2 ml) were mixed with TMB (10 mg/ml, 100 μl) and further added with H_2O_2 to the reaction solution with a final concentration of 1 mM. The absorbance at 652 nm of the reaction mixture was measured after H_2O_2 addition at 37°C for 5 min under a dark condition.

Detection of NO generation: The enzyme-mediated generation of NO by EMB-Hu was conducted in PBS (pH 7.4) containing 500 μg/mL Fe_3O_4 NPs and 150 μg/mL Hu. The enzyme-mediated reaction was initiated by adding H_2O_2 (100 μM). The generation of NO was quantified using the Greiss reagent colorimetric assay (Beyotime Biotechnology) according to the manufacturer's instructions.

Cell cytotoxicity study: First, human oral keratinocytes (HOK) were grown in 96-well plates at a concentration of 10^4 cells/well for 24 h. Then, different concentrations of EMB-Hu were incubated with HOK for 24 h. Finally, the cell viability of HOK was measured by an LDH cytotoxicity colorimetric assay kit (BioVision) according to the manufacturer's instructions.

Hemolysis assays: Red blood cells (RBCs) were collected from Balb/c mouse whole blood and isolated by centrifugation at 2000 rpm for 15 min at 4°C. After being washed with saline 3 times, the RBCs (2%) were incubated with different concentrations of MB-Hu and EMB-Hu for 12 h. RBCs incubated with H_2O and saline were used as positive control and negative control. Finally, after isolating MBs with a magnet, the absorbance of the supernatant was measured at 540 nm. The ratio of hemolysis of EMB-Hu was calculated by the following formula:

Hemolysis ratio (%) = $(A_S - A_N) / (A_P - A_N) \times 100\%$.

Where A_S represents the absorbance of the RBC solution after incubation with samples, A_N represents the absorbance of the RBC solution after incubation with saline, and A_P represents the absorbance of the RBC solution after incubation with H_2O.

Phagocytosis effect of macrophages on MRSA biofilms: The MRSA dispersed in LB (containing 1% glucose) was added to a 12-well culture plate (10^7 CFU/mL, 500 μL/well) and grown for 48 h to form MRSA biofilms. The MRSA biofilms were then stained with SYTO 9 (Thermo Fisher Scientific) for 2 h and washed with saline three times. The MRSA biofilms were then incubated with Hu, EFe + Hu, EMBs, and EMB-Hu (Fe_3O_4 NPs: 0.5 mg/mL; Hu: 0.15 mg/mL, 500 μL), stimulated with US (1 MHz, 0.5 W/cm^2, 50% amplitude, 10 min) for US treated groups, added to RAW 264.7 (10^7 cell/well), and cultured for 24 h. After that, all samples were collected, centrifuged at 1000 rpm for 3 min to remove the supernatant, and stained with PerCP-Cy5.5 anti-mouse F4/80 antibody (BioLegend, catalog no. 123125, clone BM8) for gating RAW 264.7, and finally measured by using an SH800 cell sorter (SONY, Japan) and analyzed through FlowJo software.

Supplementary Figures

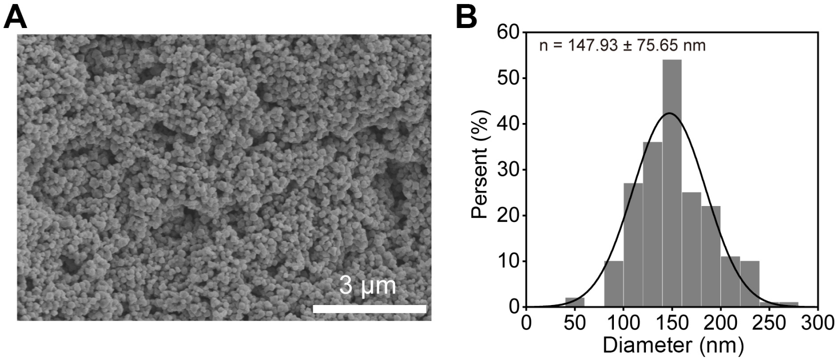

Figure S1. Characterization of Fe_3O_4 NPs. **A**) SEM image and **B**) diameter distribution of Fe_3O_4 NPs (n = 200).

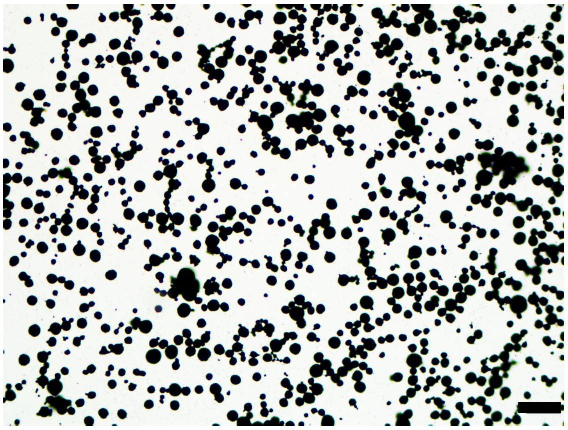

Figure S2. Bright-field microscopy image of MB-Hu. Scale bar is 20 μm.

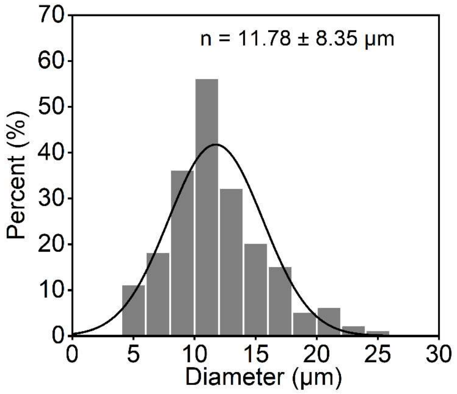

Figure S3. The diameter distribution of MB-Hu (n = 200).

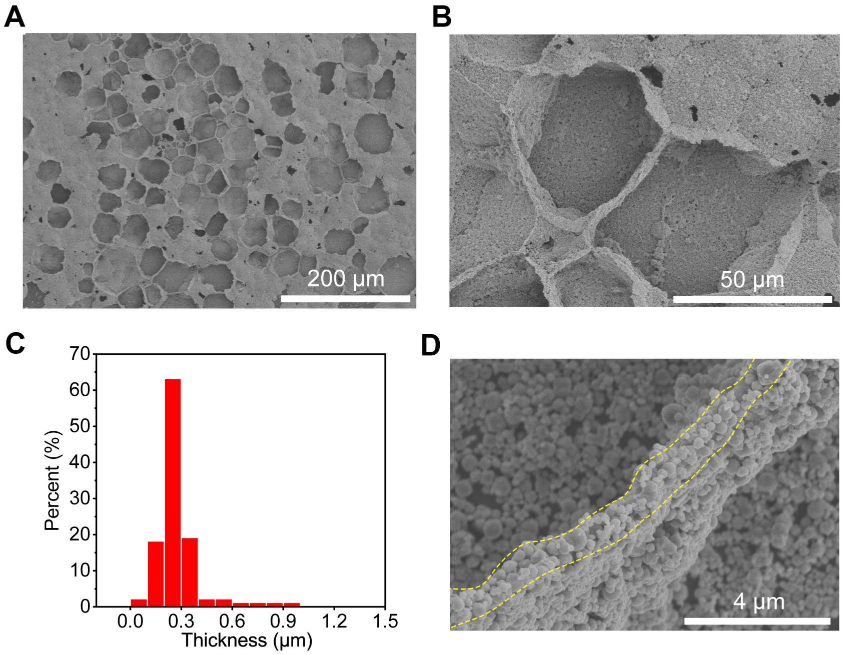

Figure S4. Characterization of EMB-Hu. **A**) SEM images of the broken EMB-Hu with low magnification and **B**) high magnification. **C**) Thickness distribution of Fe_3O_4 NPs layers in MB-Hu (*n* = 200). **D**) The edge images of broken MB-Pip.

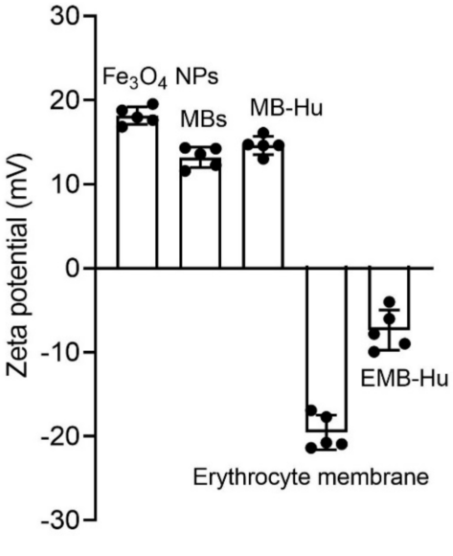

Figure S5. Zeta potential of Fe_3O_4 NPs, MBs, MB-Hu, erythrocyte membrane, and EMB-Hu (*n* = 5, means ± s.d.).

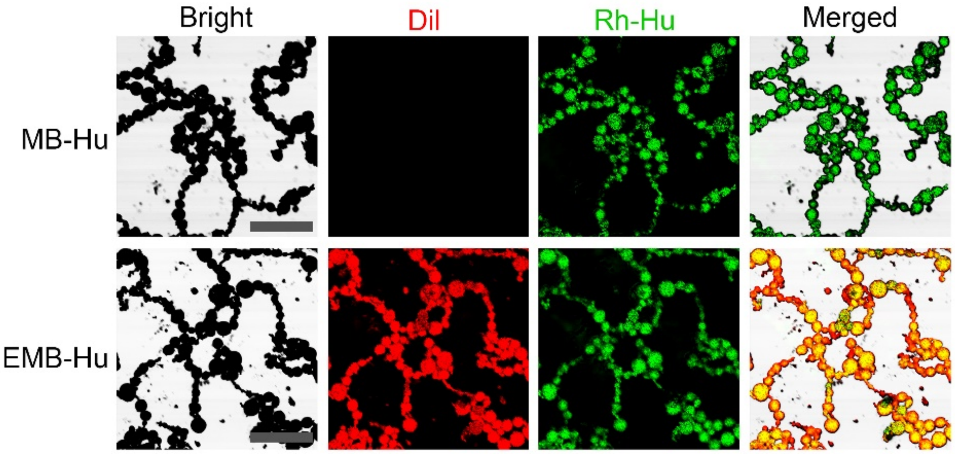

Figure S6. CLSM images of MB-Hu and EMB-Hu stained by DiI (red) and Rh-Hu (green). Scale bar is 50 μm.

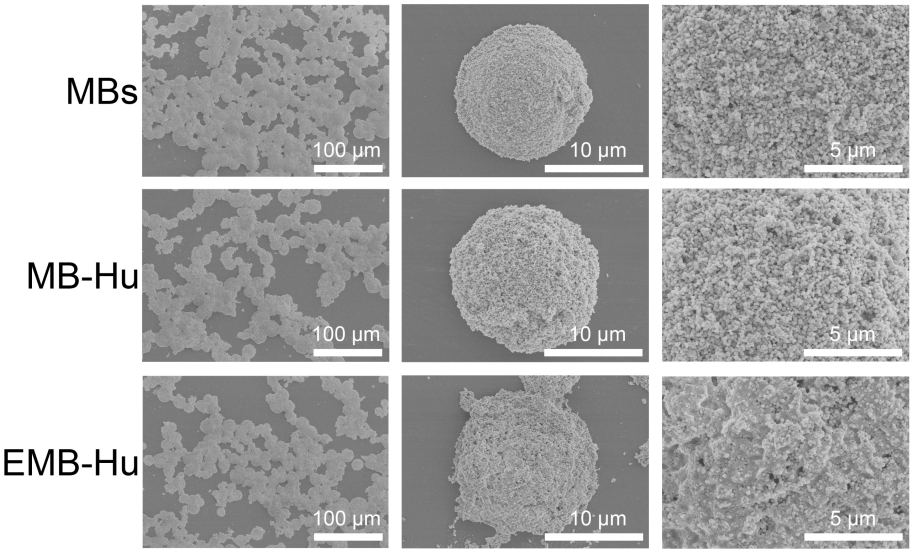

Figure S7. SEM images of MBs, MB-Hu, and EMB-Hu.

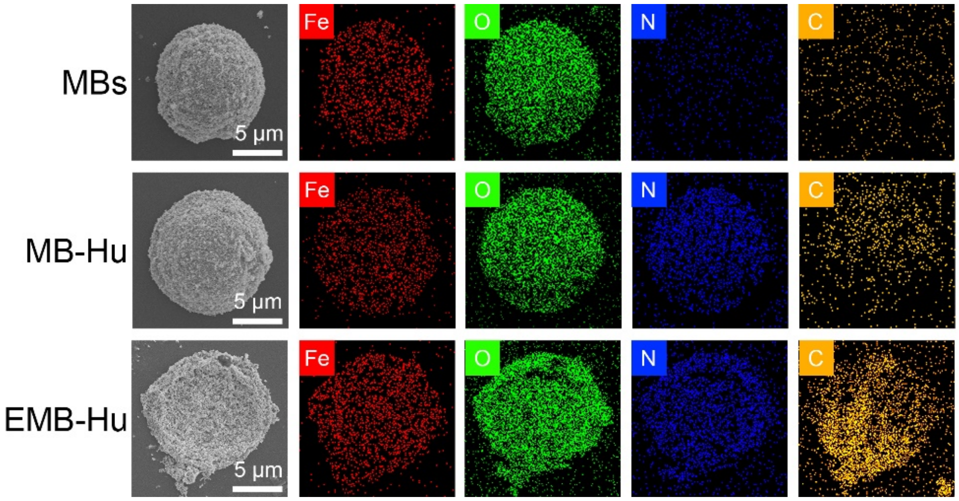

Figure S8. Elemental mapping images of MBs, MB-Hu, and EMB-Hu.

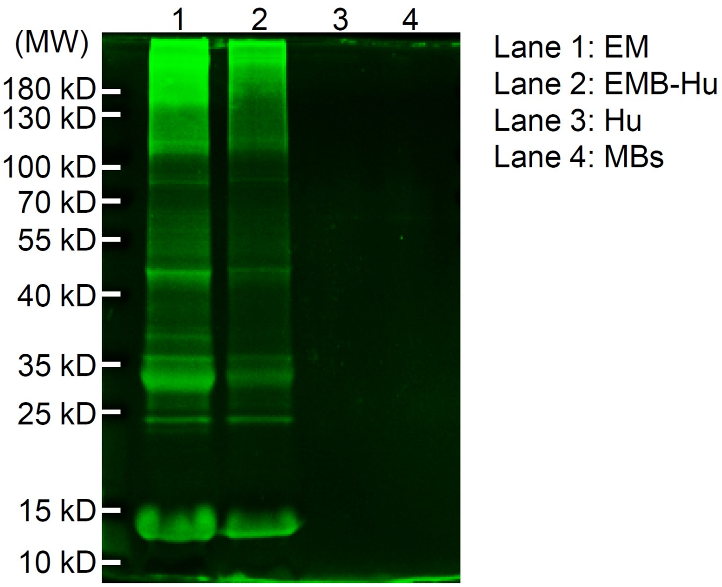

Figure S9. Protein electrophoresis of EM, EMB-Hu, Hu, and MBs (EM: erythrocyte membrane fragments). The gel was stained with One-Step Lumitein^{TM} UV (Thermo Fisher). All samples were digested with 0.5 M NaCl before electrophoresis.

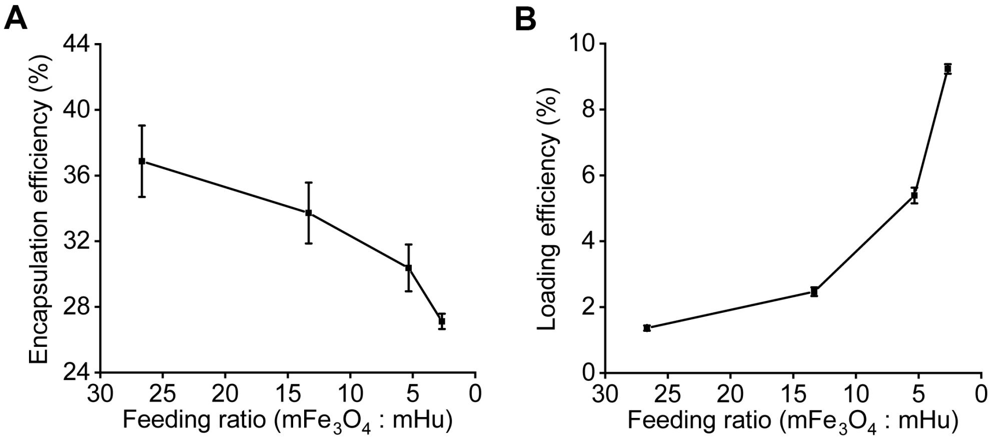

Figure S10. Loading of Hu in MB-Hu. **A**) The encapsulation efficiency and **B**) loading efficiency of Hu in MB-Hu under different feeding ratios of Fe_3O_4 NPs and Hu (n = 3, means ± s.d.).

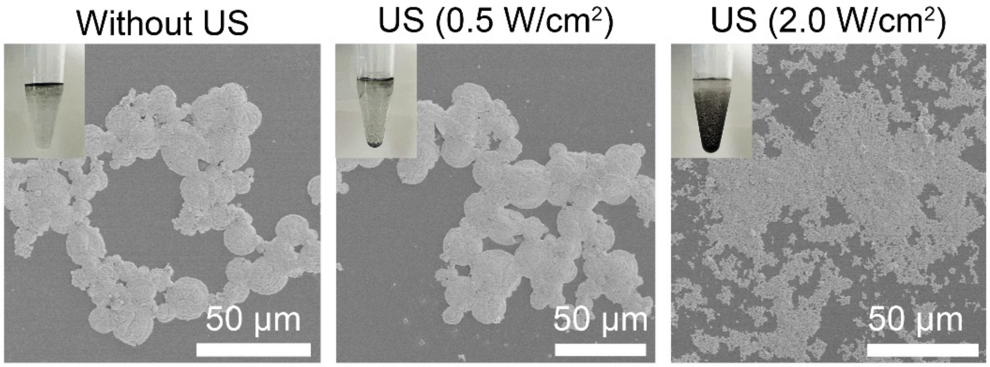

Figure S11. Photographs (insert) and SEM images of EMB-Hu under different conditions (US: 1 MHz, 50% amplitude, 10 min).

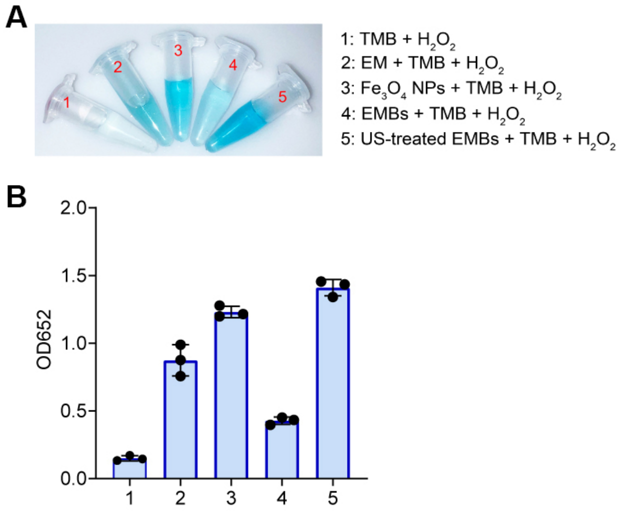

Figure S12. Peroxidase-like catalytic activity of EMB-Hu. **A**) Photographs of TMB solutions under different experimental conditions. **B**) Peroxidase-like catalytic activity of different agents (EM: erythrocyte membrane fragments) (US: 1 MHz, 0.5 W/cm^2, 50% amplitude, 10 min) by using TMB as a substrate (n = 3, means ± s.d.).

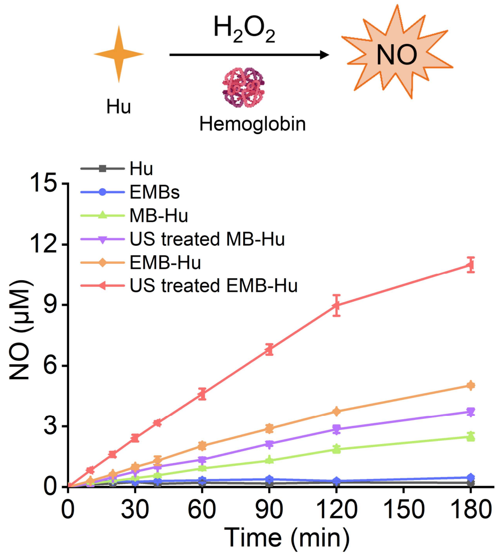

Figure S13. Generation of NO under different incubation conditions in the presence of H_2O_2 (100 μM) (US: 1 MHz, 0.5 W/cm^2, 50% amplitude, 10 min). The concentrations of Fe_3O_4 NPs and Hu were 500 μg/mL and 150 μg/mL, respectively (n

= 3, means ± s.d.).

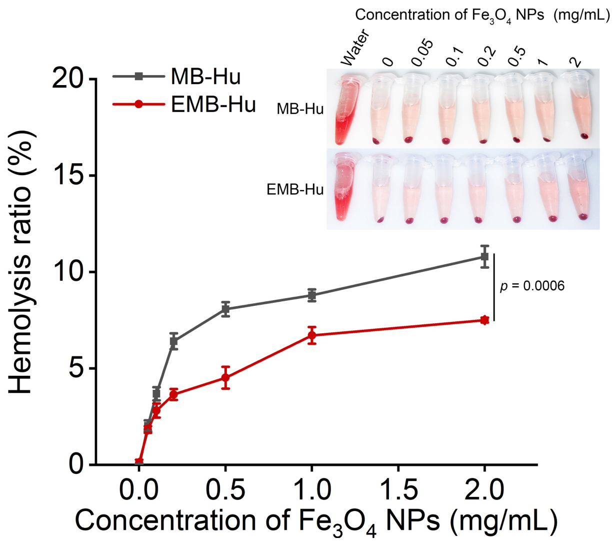

Figure S14. Hemolysis ratio of fresh mouse red blood cells (RBCs) incubated with MBP-Pip for 12 h. Inset: Photos of RBCs incubated with water, PBS, and different concentrations of MB-Hu and EMB-Pip for 12 h (n = 3, means ± s.d.). Statistical significance was analyzed via one-way analysis of variance (ANOVA) with Tukey's post-hoc test.

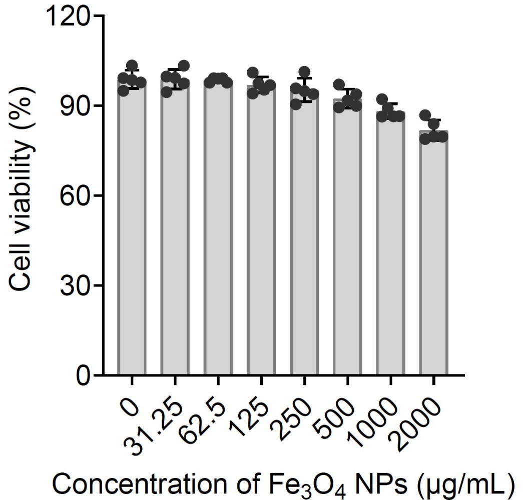

Figure S15. Viability of human oral keratinocytes (HOK) after incubation with EMB-Hu at different concentrations (n = 3, means ± s.d.).

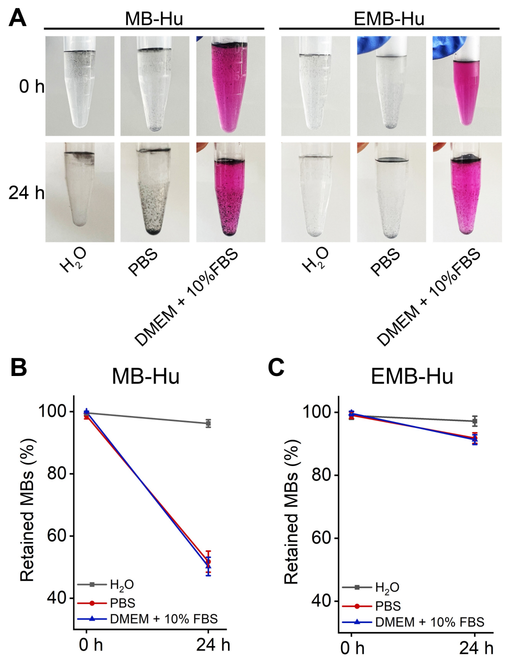

Figure S16. Stability of EMB-Hu. **A**) Photographs of MB-Hu and EMB-Hu incubated with H_2O, PBS, and DMEM with 10% FBS for different times. **B**) Retained MB-Hu and **C**) EMB-Hu after incubation with H_2O, PBS, and DMEM with 10% FBS for different times (n = 3, means ± s.d.).

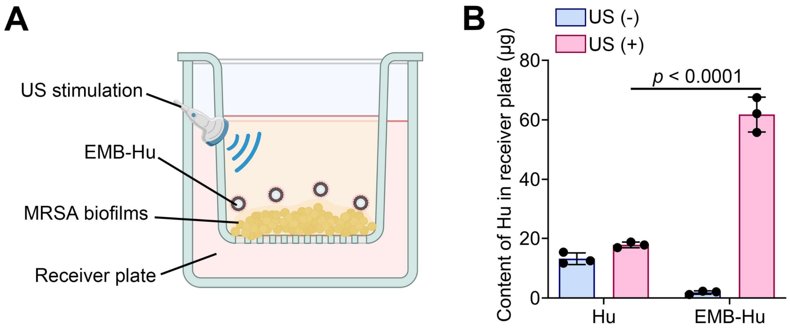

Figure S17. US-stimulated Hu penetration in MRSA biofilm. **A**) Schematic characterization of the US-stimulated Hu penetration by EMB-Hu in MRSA biofilms by using a Transwell assay. **B**) The amount of Hu in the receiver plate after various treatments (Fe_3O_4 NPs: 500 μg/mL; Hu: 150 μg/mL; H_2O_2: 100 μM; US: 1 MHz, 0.5 W/cm^2, 50% amplitude, 10 min) (n = 3, means ± s.d.). Statistical significance was

analyzed via two-way analysis of variance (ANOVA) with Tukey's post-hoc test.

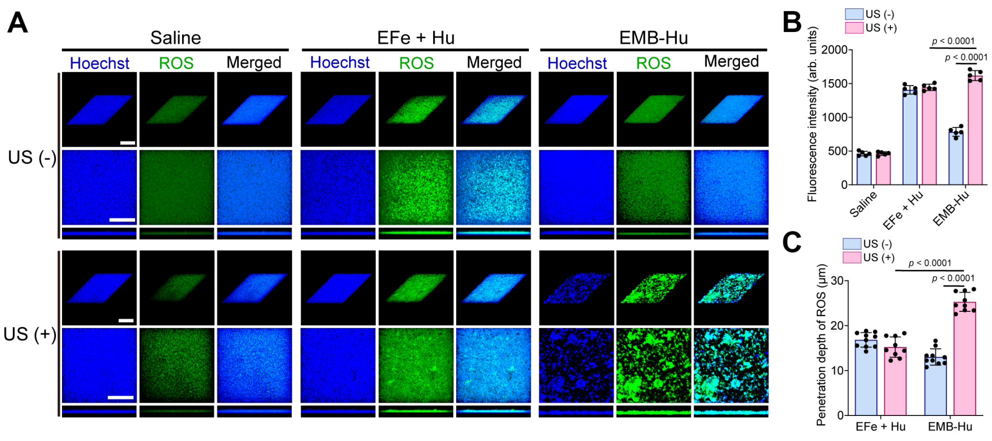

Figure S18. US-stimulated ROS penetration in MRSA biofilms. **A**) 3D CLSM images of MRSA biofilm stained by Hoechst and DCFH-DA (ROS detection probe) after various treatments (Fe_3O_4 NPs: 500 μg/mL; Hu: 150 μg/mL; H_2O_2: 100 μM; US: 1 MHz, 0.5 W/cm^2, 50% amplitude, 10 min). Scale bar is 200 μm. **B**) Fluorescence intensity (n = 5) and **C**) penetration depth (n = 10) of ROS in MRSA biofilms after various treatments calculated from (**A**) (means ± s.d.). Statistical significance was analyzed via two-way analysis of variance (ANOVA) with Tukey's post-hoc test.

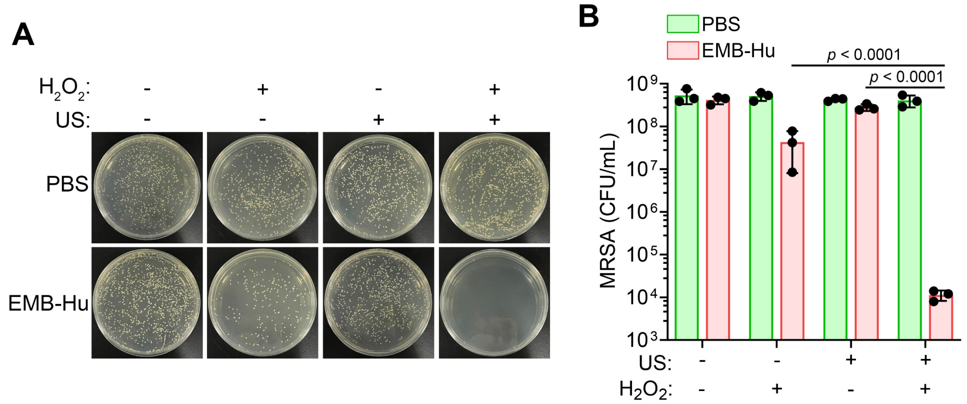

Figure S19. Anti-MRSA biofilm efficiency of EMB-Hu. **A**) Photographs of MRSA colonies plate and **B**) the number of live MRSA within MRSA biofilm after various treatments (Fe_3O_4 NPs: 500 μg/mL; Hu: 150 μg/mL; H_2O_2: 100 μM; US: 1 MHz, 0.5 W/cm^2, 50% amplitude, 10 min) (n = 3, means ± s.d.). Statistical significance was

analyzed via two-way analysis of variance (ANOVA) with Tukey's post-hoc test.

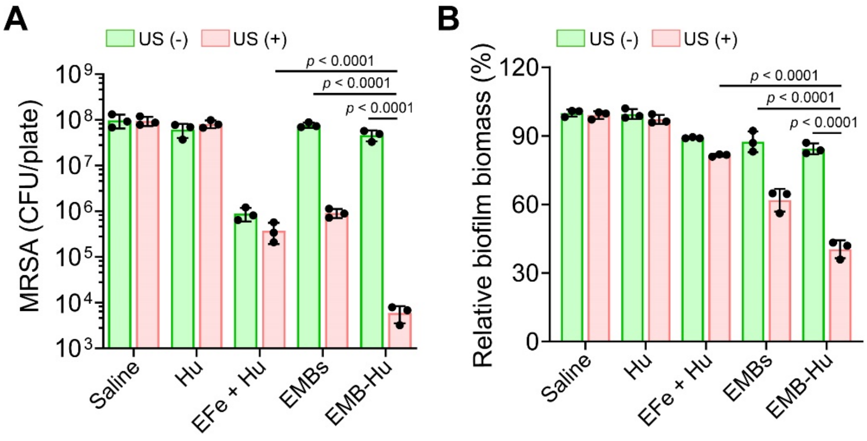

Figure S20. Treatment of MRSA biofilm growth in titanium plate *in vitro*. **A**) the number of live bacteria within MRSA biofilms growth in titanium plate in different groups with the presence of H_2O_2 (Fe_3O_4 NPs: 500 μg/mL; Hu: 150 μg/mL; H_2O_2: 100 μM; US: 1 MHz, 0.5 W/cm^2, 50% amplitude, 10 min) (n = 3, means ± s.d.). **B**) Relative biofilm biomass biofilm growth in titanium plate with the presence of H_2O_2 in different groups (n = 3, means ± s.d.).

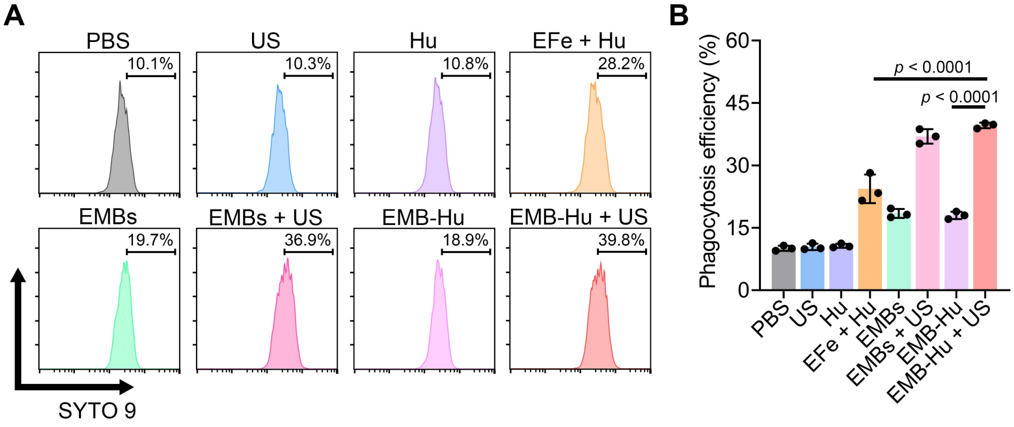

Figure S21. Phagocytosis effect of RAW 264.7 cells on MRSA biofilms. **A**) Representative flow cytometry plots and **B**) quantification of phagocytic clearance of MRSA within biofilms by RAW264.7 macrophages (F4/80$^+$) with the indicated treatments. (*n* = 3, means ± s.d.). Statistical significance was analyzed via one-way analysis of variance (ANOVA) with Tukey's post-hoc test.

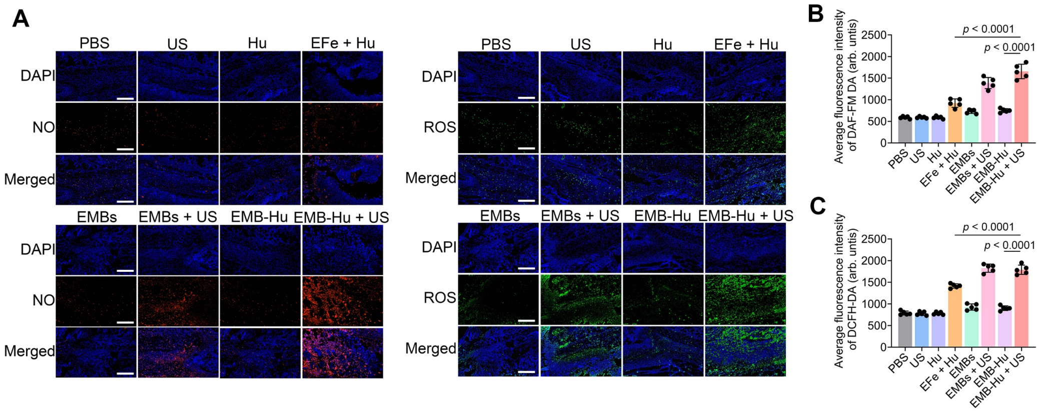

Figure S22. Penetration of ROS and NO in infected tissues. **A**) Fluorescence microscope images of infected tissue slices after various treatments stained by DAF-FM DA (red, NO detection probe) and **B**) DCFH-DA (green, ROS detection probe). Scale bar is 200 μm. **C**) The average fluorescence intensity of DAF-FM DA and **D**) DCFH-DA (n = 5, means ± s.d.). Statistical significance was analyzed via one-way analysis of variance (ANOVA) with Tukey's post-hoc test.

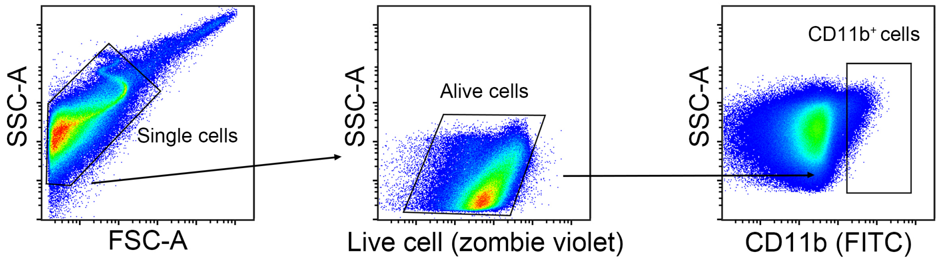

Figure S23. A gate drawn around the cell collected from infected tissues. Live CD11b$^+$ cell analysis was carried out at this gate.

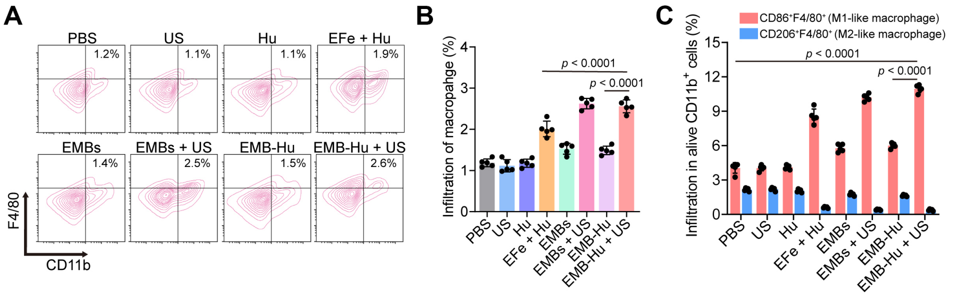

Figure S24. Polarization of macrophages in infected tissues. **A**) Flow cytometry analysis of macrophages ($CD11b^+F4/80^+$) in infected tissue after various treatments for 3 days. **B**) Infiltration of macrophages in infected tissue after various treatments for 3 days (n = 5, means ± s.d.). **C**) Polarization of macrophages (M1-like macrophages: $CD11b^+F4/80^+CD86^+$ cells; M2-like macrophages: $CD11b^+F4/80^+CD206^+$ cells) in infected tissue after various treatments for 3 days (n = 5, means ± s.d.). Statistical significance was analyzed via (**B**) one-way and (**C**) two-way analysis of variance (ANOVA) with Tukey's post-hoc test.

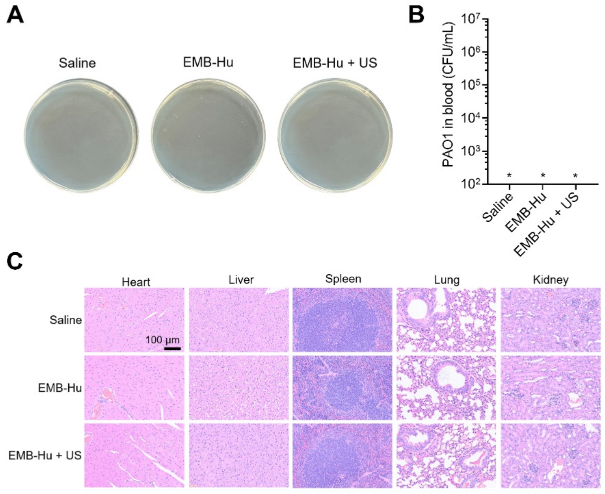

Figure S25. Toxicity study of infected mice after treated by EMB-Hu with US stimulation. **A**) Photographs of MRSA colonies plate and **B**) the number of live MRSA in blood of infected mice after various treatments for 24 h (n = 5, means ± s.d.). **C**) H&E staining images of organ sections (heart, liver, spleen, lung, and kidney) from infected mice after various treatments for 24 h. The asterisk represents the

number of PAO1 is less than the limit of detection (10^2 CFU).

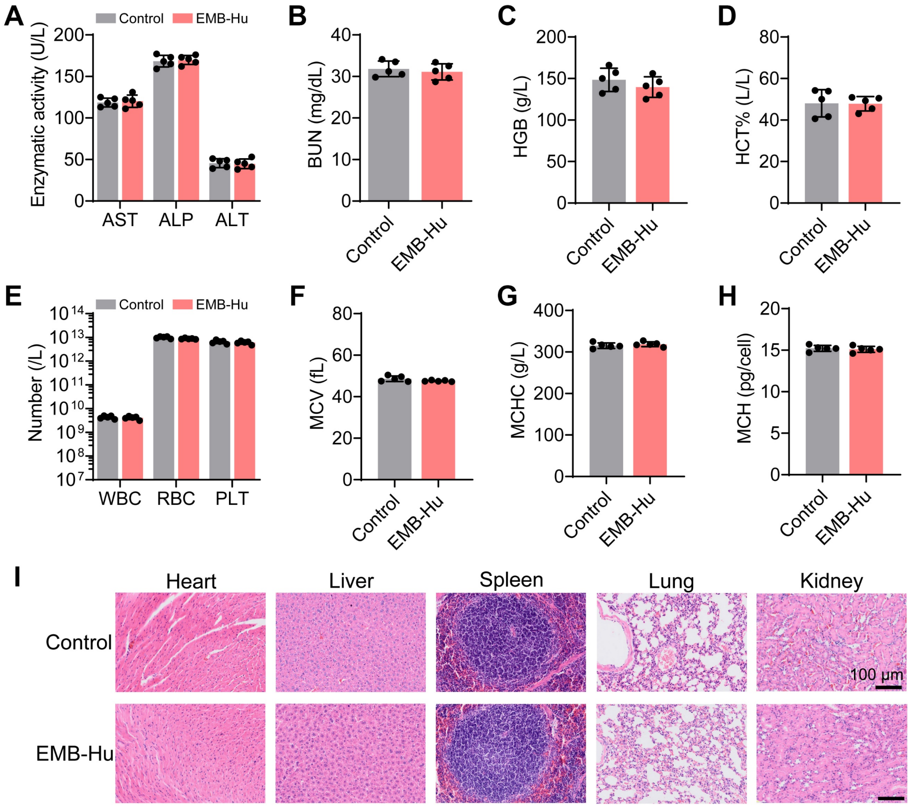

Figure S26. Toxicity evaluation of EMB-Hu *in vivo*. **A**) Aspartate aminotransferase (AST), alanine aminotransferase (ALT), alkaline phosphatase (ALP), **B**) blood urea nitrogen (BUN), **C**) hemoglobin (HGB), **D**) hematocrit (HCT), **E**) white blood cells (WBC), red blood cells (RBC), platelets (PLT), **F**) mean corpuscular volume (MCV), **G**) mean corpuscular hemoglobin concentration (MCHC), and **H**) mean corpuscular hemoglobin (MCH) levels in the blood at 21 d post-injection of PBS (Control) or EMB-Hu (dose of Hu = 6 mg/kg, dose of Fe_3O_4 = 20 mg/kg) (n = 5, means ± s.d.). **I**) H&E staining images of major organ sections (heart, liver, spleen, lung, and kidney) from mice at 21 d post-injection of PBS (Control) or EMB-Hu.
